# Ca_V_1.3 channel clusters characterized by live-cell and isolated plasma membrane nanoscopy

**DOI:** 10.1038/s42003-024-06313-3

**Published:** 2024-05-23

**Authors:** Niko Schwenzer, Nikolas K. Teiwes, Tobias Kohl, Celine Pohl, Michelle J. Giller, Stephan E. Lehnart, Claudia Steinem

**Affiliations:** 1 Department of Cardiology and Pneumology, University Medical Center Göttingen, Robert-Koch-Str. 40, 37075 Göttingen, Germany; 2 Cellular Biophysics and Translational Cardiology Section, Heart Research Center Göttingen, University Medical Center Göttingen, Robert‑Koch‑Str. 42a, 37075 Göttingen, Germany; 3 Cluster of Excellence “Multiscale Bioimaging: from Molecular Machines to Networks of Excitable Cells” (MBExC 2067), University of Göttingen, 37073 Göttingen, Germany; 4 Georg-August Universität, Institut für Organische und Biomolekulare Chemie, Tammannstr. 2, 37077 Göttingen, Germany; 5 DZHK (German Centre for Cardiovascular Research), partner site Göttingen, Robert-Koch-Str. 40, 37075 Göttingen, Germany; 6 Collaborative Research Center SFB 1190 “Compartmental Gates and Contact Sites in Cells”, University of Göttingen, Humboldtallee 23, 37073 Göttingen, Germany; 7 Max-Planck-Institut für Dynamik und Selbstorganisation, Am Fassberg 17, 37077 Göttingen, Germany

**Keywords:** Nanoscale biophysics, Membranes, Fluorescence imaging

## Abstract

A key player of excitable cells in the heart and brain is the L-type calcium channel Ca_V_1.3. In the heart, it is required for voltage-dependent Ca^2+^-signaling, i.e., for controlling and modulating atrial cardiomyocyte excitation-contraction coupling. The clustering of Ca_V_1.3 in functionally relevant channel multimers has not been addressed due to a lack of stoichiometric labeling combined with high-resolution imaging. Here, we developed a HaloTag-labeling strategy to visualize and quantify Ca_V_1.3 clusters using STED nanoscopy to address the questions of cluster size and intra-cluster channel density. Channel clusters were identified in the plasma membrane of transfected live HEK293 cells as well as in giant plasma membrane vesicles derived from these cells that were spread on modified glass support to obtain supported plasma membrane bilayers (SPMBs). A small fraction of the channel clusters was colocalized with early and recycling endosomes at the membranes. STED nanoscopy in conjunction with live-cell and SPMB imaging enabled us to quantify Ca_V_1.3 cluster sizes and their molecular density revealing significantly lower channel densities than expected for dense channel packing. Ca_V_1.3 channel cluster size and molecular density were increased in SPMBs after treatment of the cells with the sympathomimetic compound isoprenaline, suggesting a regulated channel cluster condensation mechanism.

## Abstract (web summary)

The authors develop a HaloTag-labeling strategy to quantify the clustering of the L-type calcium channel CaV1.3 using live-cell STED nanoscopy.

## Introduction

Voltage-gated Ca^2+^ (Ca_V_) channels are expressed in excitable as well as non-excitable cells^1^. Among the different members of the Ca_V_ channel family, the Ca_V_1 subfamily (Ca_V_1.1–Ca_V_1.4) constitutes the so-called L-type calcium channels showing long (L) lasting currents^2^. Ca_V_1 channels are expressed in cardiac myocytes and pacemaker cells, nerves, endocrine cells, the inner ear, and the retina, where their specific physiological roles correlate tightly with their distribution at the cell membrane^3,4^. Over the years, valuable knowledge has been gathered about Ca_V_1 channels and their distribution at the plasma membrane and a picture has evolved that these channels may multimerize in dense clusters^5^. Indeed, the clustering of Ca_V_1 channels is necessary for the local amplification of Ca^2+^-influx.

To be able to investigate and quantify individual Ca_V_1 channel clusters in live cells with super-resolution microscopy techniques, appropriate labeling strategies are required. Most studies on Ca_V_1 channels relied on indirect immunostaining strategies with the drawback of artifacts arising from the chemical fixation and an indirect, spatially distorting immunodetection^6–11^. Alternatives to immunofluorescence staining are generally based on tagging the Ca_V_1 channels with fluorescent proteins^12,13^. Using this approach, Ca_V_1.2 clusters have been examined using super-resolution microscopy on fixed cells^14–16^. For live-cell imaging, Conrad et al. reported on the labeling of Ca_V_1.2 on the cell surface of HEK293 cells using a HaloTag^12^. They recorded the postendocytic trafficking of Ca_V_1.2 via spinning disc confocal microscopy. However, this method does not provide the required resolution to resolve Ca_V_1 clusters at the nanoscale, i.e., individual channels with a diameter of 10 nm.

The HaloTag-based approach appears to be well suited to investigate and quantify Ca_V_1 channel clusters in live cells using super-resolution microscopy because it is stoichiometric, allowing for 1:1 labeling and channel counting. Here, we focused our attention on the Ca_V_1.3 channel as it is generally understudied, while its role in the atria and inner ear appears to be of paramount significance^17^. Both Ca_V_1.3 and Ca_V_1.2 are expressed in many of the same tissues, including the brain, heart, and endocrine glands^2^. However, the Ca_V_1.3 channel exclusively contributes to the cardiac excitation-contraction coupling in atrial cardiomyocytes. Based on Ca_V_1.3 channel Ca^2+^ influx, pace-making in the sinoatrial node leads to activation of cardiomyocyte excitation-contraction coupling and rapid shortening of atrial in contrast to ventricular cardiomyocytes^18,19^. Similar to its homolog Ca_V_1.2, Ca_V_1.3 associates into channel clusters that modulate channel function and facilitate coordinated local Ca^2+^-influx^5,20–22^. However, the size and structure of individual clusters, the number of channels within a cluster, and their packing density have not been elucidated owing to the lack of an appropriate high-resolution imaging strategy.

Here, we describe a HaloTag-Ca_V_1.3 construct applicable for stoichiometric super-resolution microscopy in living cells to investigate channel clusters quantitatively. This approach enabled us to visualize Ca_V_1.3 clusters in the plasma membrane and their endosomal colocalization in transfected live cells using STED nanoscopy. We quantified the number of channels as well as their intra-cluster density in the plasma membrane of HEK293 cells. We compared the obtained results with those gathered from isolated plasma membranes derived from living cells that further facilitated the high-resolution cluster quantification using STED nanoscopy owing to their planarity and fixation on the support. Planar plasma membranes attached to modified glass support were derived from spreading giant plasma membrane vesicles (GPMVs) obtained from living HEK293 cells. GPMVs have been shown previously to be well suited to study plasma membrane structures and dynamics, including protein clusters^23–27^. However, only a few examples are reported in the literature that demonstrate the successful spreading of GPMVs on planar support to make the membranes readily amenable for super-resolution microscopy ^28–31^.

## Results

### Halo-Ca_V_1.3 clusters at the cell surface of HEK293 cells

A HaloTag was N-terminally coupled to the human Ca_V_1.3 channel pore subunit (Halo-α_1D_). N-terminal, intracellularly located fusion tagging has been previously shown not to affect channel function^32^. A HEK293 cell line (CT6232) with an inducible expression of the pore-forming α_1D_ subunit and a constitutive expression of the accessory channel subunits α_2_δ_1_ and β_3_ was used to transiently transfect Halo-α_1D_ forming Halo-Ca_V_1.3 channel complexes, while non-tagged Ca_V_1.3^WT^ expression was used as molecular weight control since channel glycosylation may affect its gel migration. Protein synthesis and stability of full-length Halo-Ca_V_1.3 channels were validated by Western blots (Supplementary Fig. 1a). The expression of the Ca_V_1.3 subunits α_2_δ_1_ and β_3_, which are necessary for channel assembly, trafficking, and posttranslational functional regulation were confirmed as well as Ca_V_1.3^WT^, Ca_V_1.3-HA, Halo-Ca_V_1.3 and EGFP-Ca_V_1.3 α_1D_, the latter two showing a larger apparent molecular weight than Ca_V_1.3^WT^ as expected. Functional Halo-Ca_V_1.3 surface membrane expression was confirmed with electric field-evoked intracellular calcium transients. The recorded transients were similar to those recorded following expression induction of Ca_V_1.3^WT^, whereas un-transfected control cells did not show any calcium transients (Supplementary Fig. 1b). The electrically evoked calcium transients were completely abolished by the addition of the pore blocker nifedipine (10 µM). A comparison of confocal laser scanning fluorescence images revealed that the live-cell HaloTag labeling results in a substantially improved specificity and signal-to-noise ratio compared to conventional indirect immunofluorescence detection of Ca_V_1.3 following literature protocols (Supplementary Fig. 1c, d).

**Fig. 1:**
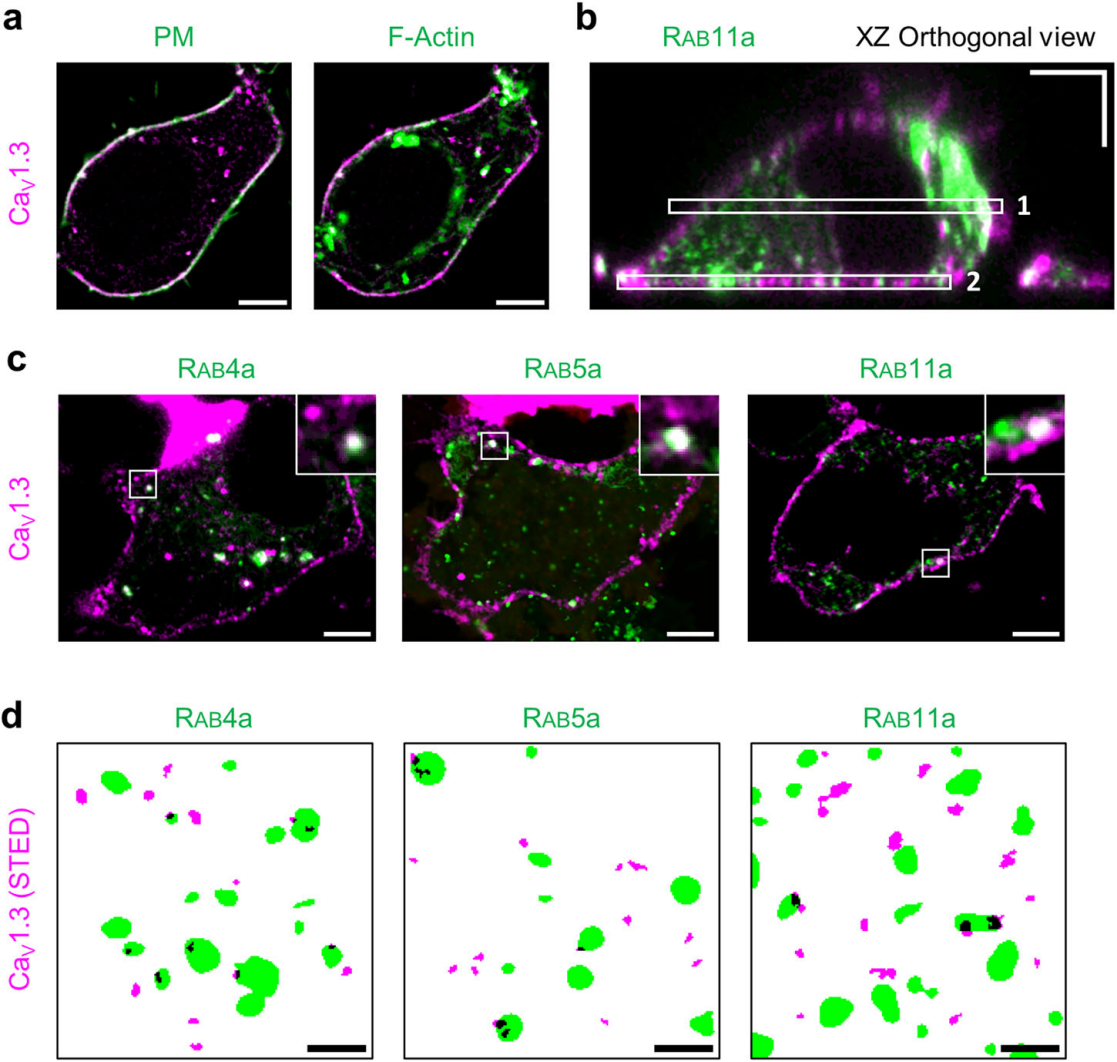
Cell surface localization and endosomal transport of Ca_V_1.3 analyzed in transiently transfected living HEK293 cells. **a** Confocal images showing an overlay of Halo-Ca_V_1.3 (JF646-HTL, magenta) with either PM (green) or F-actin signals (green) generated by three-color co-staining. **b** Orthogonal XZ image of a cell expressing Halo-Ca_V_1.3 (magenta) and GFP-Rab11a (green). Two principal imaging planes used for colocalization analysis are highlighted by white boxes: Medial cell sectioning (1) used for protein localization in (**a**, **c**), basal plane imaging (2) used for quantitative colocalization analysis in (**d**). **c** Confocal images of cells co-expressing Halo-Ca_V_1.3 with GFP-Rab4a, GFP-Rab5a, and GFP-Rab11a, respectively. **d** Representative segmentation maps of cell surface localized Halo-Ca_V_1.3 signals (magenta, STED) together with each indicated endosomal marker (green, confocal). Colocalization is shown in black. Scale bars: 5 µm (**a**–**c**), 1 µm (**d**).

Based on these results, we next addressed the question about the localization and distribution of Ca_V_1.3 in HEK293 cells. Live-cell confocal images were taken to investigate the colocalization of Halo-Ca_V_1.3 with a fluorescent marker for the plasma membrane (Fig. 1a, PM, green) and cortical F-actin (Fig. 1a, F-actin, green) showing cell surface localization. Assuming continuous, steady-state channel turnover through endosomal pools at the cell surface, we furthermore co-expressed GFP-tagged Rab4a, Rab5a, and Rab11a as established markers of the early endocytic and recycling pathways and imaged the medial (1) and basal plane (2) of orthogonal sections of HEK293 cells (Fig. 1b). Colocalization of Ca_V_1.3 with endosomes marked by either Rab4a, Rab5a, or Rab11a was observed (Fig. 1c).

To quantify Halo-Ca_V_1.3 spots colocalized to these Rab-labeled compartments, we imaged the intracellular, basal cell membrane plane (Fig. 1b(2)). Here, we exploited the possibility of imaging cell surface Halo-Ca_V_1.3 by live-cell nanoscopy using STimulated Emission Depletion (STED) nanoscopy. The HaloTag ligand (HTL) conjugate of JF646, a SiR-derived fluorogenic dye, exhibits high brightness in the far-red spectral range^33^, and can be readily combined with confocal images to detect the endosomal GFP-Rabs. Spots of Halo-Ca_V_1.3 were visualized by STED nanoscopy, which we assigned to Halo-Ca_V_1.3 nanoclusters. To determine the colocalization of these Halo-Ca_V_1.3 nanoclusters with the Rab signals, the images were filtered and segmented by automatic thresholding of specific Rab signals and Halo-Ca_V_1.3 signals to generate binary maps (Fig. 1d). These maps were used for the colocalization analysis, defining colocalized clusters as overlaps of Halo-Ca_V_1.3 and GFP-Rab signals.

After accounting for nonspecific colocalization events given by Rab-positive cell area fractions^34^, only low fractions of Halo-Ca_V_1.3 clusters were found to colocalize with endosomal populations (8.4% Rab4a, 3.8% Rab5a, 5.0% Rab11a, Supplementary Table 1). In summary, 17.2% Ca_V_1.3 clusters were colocalized with Rab-marked endosome compartments, implying indirectly that the majority of channel clusters are localized within the plasma membrane. Supporting the validity of our approach, the mean observed density of endosomal spots (Rab4a 0.19 spots/µm^2^, Rab5a 0.17 spots/µm^2^, and Rab11a 0.73 spots/µm^2^) and Ca_V_1.3 clusters (0.69 clusters/µm^2^) at the cell surface was analyzed in three independent experiments and turned out to provide the same results.

When comparing Rab-associated with unassociated (mainly plasma membrane-localized) Halo-Ca_V_1.3 clusters, we did not find a significant difference in cluster area or brightness (see analysis below), thus confirming the integrity of cluster assemblies during endosomal transport.

### Live-cell nanoscopy of Halo-Ca_V_1.3 quantifies cell-surface channel clustering

**Fig. 2:**
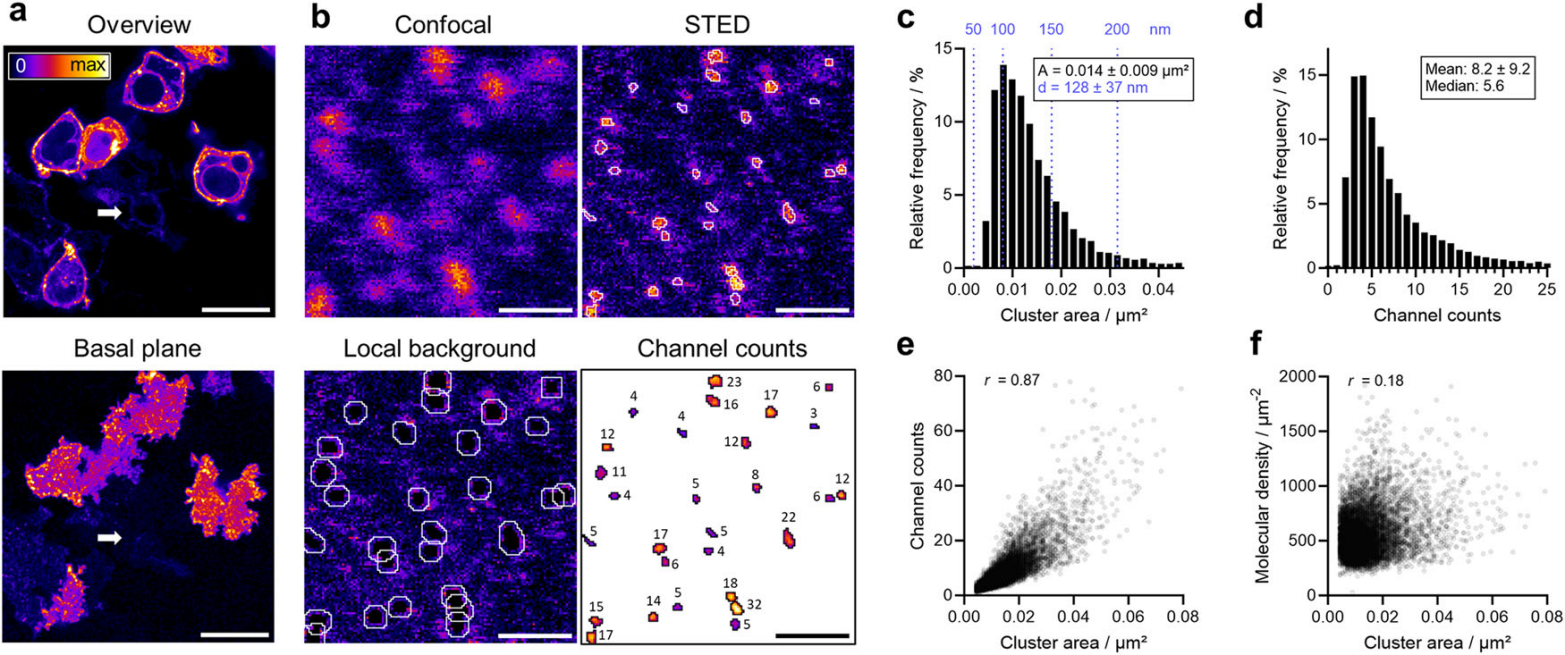
Nanoscopic cluster analysis of Halo-Ca_V_1.3 STED signals at the surface of HEK293 cells. **a** Live-cell confocal images of transiently transfected HEK293 cells show cell-surface localized signals of Halo-Ca_V_1.3 channels labeled with JF646-HTL (‘Fire’ LUT). The lower-hand image demonstrates high signal densities in the basal PM imaging plane. **b** Cell-surface STED nanoscopy revealed a clustered distribution of Halo-Ca_V_1.3 signals, as resolved by STED but not by confocal imaging. Individual cluster signals were segmented as shown by white outlines. For each cluster, the signal brightness was corrected for the local background prior to molecular counting by brightness referencing. **c** Frequency distribution of the cluster area obtained from segmented signals (*n*_clusters_ = 9459 clusters from *n*_cell_ = 75). The values in blue provide the corresponding diameter of the clusters assuming a round cluster shape. The figure legend shows the mean and s.d. for the cluster area and diameter. **d** Frequency distribution of fluorescent channel counts within the segmented cluster signals as determined by brightness referencing. **e** Scatter graph showing the relationship between fluorescent channel counts and cluster area. The correlation was quantified by Spearman’s *r* = 0.87 (*p* < 0.0001). **f** Scatter graph of the molecular density and cluster area with *r* = 0.18 (*p* < 0.0001). Scale bars: 20 µm (**a**), 1 µm (**b**).

The HaloTag ligand conjugate of JF646 enabled us to investigate the Ca_V_1.3 channel clusters in detail by quantitative live-cell STED nanoscopy. For an overview, confocal images were first taken with the Ca_V_1.3 signals at the cell surface. Un-transfected HEK293 cells could be readily distinguished as they showed negligible fluorescent signals (Fig. 2a, arrow), confirming the highly specific labeling. To selectively image individual Ca_V_1.3 clusters localized to the plasma membrane, the basal plane of the cell was used (see Fig. 1b(2)), as this section provides the largest in-frame quasi-planar density of Ca_V_1.3 signals. STED nanoscopy readily resolved the fluorescent spots in the basal plane assigned as individual Halo-Ca_V_1.3 channel clusters, which could not be resolved by confocal imaging (Fig. 2b).

Quantitative analysis of the STED images was applied to segment signal patterns into clusters and to determine their size, shape, and brightness. In the plasma membrane of the living cells, we found a mean cluster density of 0.69 ± 0.25 clusters/µm^2^. The mean area of segmented Ca_V_1.3 clusters was 0.014 ± 0.009 µm² (mean ± s.d. | median: 0.012) (Fig. 2c). This corresponds to a diameter of 128 ± 37 nm for predominantly circular clusters (roundness 0.68 ± 0.15). Largely asymmetric clusters, e.g., linear or rectangular arrangements, were not detected, and neither were large cluster assemblies like at the presynaptic membrane in inner hair cells^21^. The STED images enabled us to determine simultaneously the photon counts within each cluster, which report on the number of dye-labeled Halo-Ca_V_1.3 channels per cluster (channel counts) based on brightness referencing (Supplementary Fig. 2a–c)^35,36^. This approach was validated by stepwise photobleaching experiments (Supplementary Fig. 2d–h). From this analysis, we determined a mean value of 8 ± 9 labeled Ca_V_1.3 channels per cluster (Fig. 2d) and a median of 5.6. 79% of the channel clusters contained 10 or fewer channels.

The channel counts were well correlated with the cluster area (Spearman’s *r* = 0.87, Fig. 2e), suggesting that the molecular density within the clusters is similar among the channel clusters and independent of the cluster size. We further confirmed this notion by calculating the molecular density for each cluster as the ratio of channel counts and area. No clear dependence of the molecular density on the cluster area can be observed (Spearman’s *r* = 0.18, Fig. 2f). A mean intra-cluster channel density of 552 ± 217 µm^−2^ was found. Owing to the convolution with the STED point spread function near the resolution limit (Supplementary Fig. 3a, b), an overestimation of the cluster area is conceivable. To investigate whether the molecular density is influenced by this convolution, we calculated the molecular density using only channel clusters with an area larger than 0.015 µm² (representing 20% of the data), resulting still in a molecular density of 743 ± 283 µm^−^². As an upper bound (95th percentile), we found 945 µm^−^². These data indicate that the mean molecular density of Ca_V_1.3 clusters in live-cell membranes is at least one order of magnitude smaller than the theoretical molecular density of 10,000 channels/µm^2^ calculated by taking the lateral channel dimensions of 10 × 10 nm^2^ of the Ca_V_1.3 channels into account^37^.

### Comparison of Halo-Ca_V_1.3 clusters in cells and SPMBs probed by STED nanoscopy

**Fig. 3:**
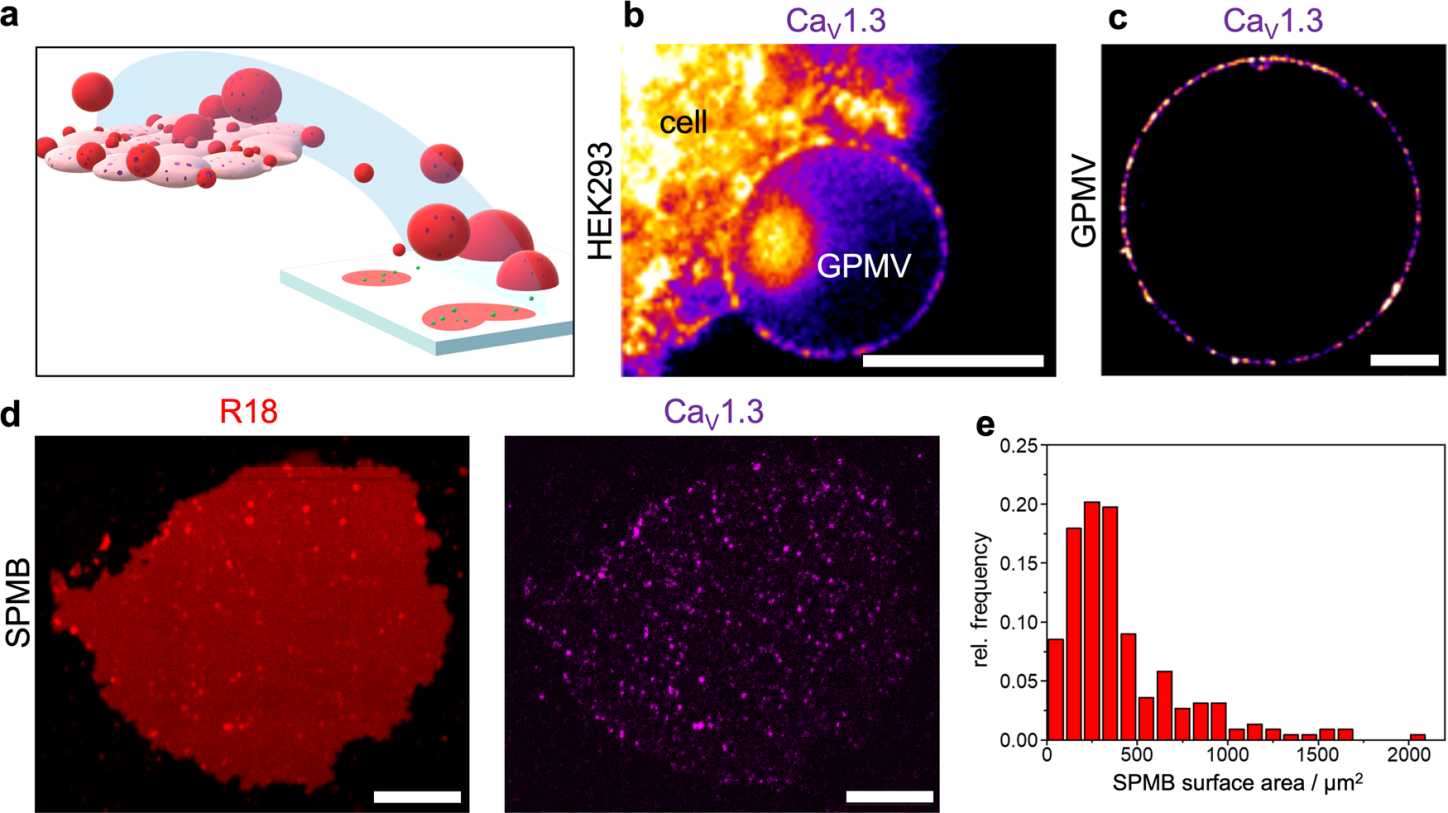
Transfer of Halo-Ca_V_1.3 channel clusters from GPMVs derived from HEK293 cells to supported plasma membrane bilayers (SPMBs). **a** Schematics of the production of GPMVs derived from cells and the spreading of GPMVs on a solid support. **b** Confocal image of the Halo-Ca_V_1.3 signal (‘Fire’ LUT) showing a GPMV attached to a HEK293 cell. **c** Upright STED image resolving spot-like fluorescent Halo-Ca_V_1.3 clusters in the detached GPMV. **d** Upright STED image of a supported plasma membrane bilayer (SPMB) obtained by spreading a GPMV on soda lime-glass (left image: R18, red; right image: Halo-Ca_V_1.3, magenta). **e** Distribution of the SPMB surface areas with a mean area of 410 ± 340 µm² (mean ± s.d.) (*n*_SPMB_ = 232). Scale bars: 5 µm (**b**), 10 µm (**c**, **d**).

Parameters that might influence the quantitative Halo-Ca_V_1.3 analysis are the mobility of the channel clusters in the basal membrane of HEK293 cells and the background arising from the cell itself. To improve the imaging sensitivity and resolution, we aimed at producing giant plasma membrane vesicles (GPMVs) that can be spread onto a solid support to form supported plasma membrane bilayers (SPMBs) (Fig. 3a). SPMBs are expected to reduce signals from the cell body and provide a planar geometry that is readily accessible by high-resolution fluorescence microscopy with low out-of-focus light, which enhances sample imaging sensitivity and resolution. It is, moreover, conceivable that the membrane structures, particularly Ca_V_1.3 channel clusters, become less mobile attached to a solid support.

To achieve this goal, we first generated GPMVs from Halo-Ca_V_1.3 transfected HEK293 cells^29^. Fluorescence images show that Halo-Ca_V_1.3 clusters were transferred from the plasma membrane of the cell into the growing GPMV membrane (Fig. 3b) and the detached GPMVs (Fig. 3c). Owing to the non-planarity of the GPMV membrane and the mobility of the Ca_V_1.3 clusters, they could only be imaged within one image plane with sufficient resolution. To obtain the required planarity of the plasma membranes with reduced mobility of the clusters, the GPMVs were spread on solid support leading to SPMBs.

The general attempt to spread GPMVs on a glass substrate (soda lime glass) treated with oxygen plasma was successful. Similar to the GPMV spreading on SiO_2_ surfaces, rather round membrane patches were observed in the fluorescence micrographs (Fig. 3d, left image) with a size distribution of 410 ± 340 µm² (Fig. 3e) resembling the area of the spread GPMVs with the intracellular membrane leaflet facing the solution (Supplementary Fig. 4). Figure 3d (right image) shows that the Ca_V_1.3 clusters are successfully transferred from the GPMV to the SPMB. However, the formation of SPMBs on soda lime glass did not allow to perform STED using the inverted microscope setup which is required to perform quantitative imaging.

To image SPMBs derived from GPMVs using a high numerical aperture (NA) inverted STED microscope as used for HEK293 cell imaging, a thin borosilicate glass is required. Borosilicate glass is used for high-NA microscopy due to its chemical inertness, low autofluorescence, and availability in a defined thickness (170 ± 5 µm), ensuring a defined optical path between the immersion oil and sample. Attempts to spread GPMVs on borosilicate glass, however, failed. On oxygen plasma-treated borosilicate glass, the GPMVs derived from HEK293 cells attached to the glass surface but did not spread to form SPMBs. To determine the parameters that are required for the successful spreading of GPMVs on a surface, we analyzed the hydrophilicity (Supplementary Table 2) and the surface roughness (Supplementary Fig. 5) of three different substrates, namely silicon dioxide, soda lime glass, and borosilicate glass. Previously, we have shown that GPMVs can be spread on silicon dioxide after oxygen plasma treatment. SiO_2_ wafers show a surface tension of *γ*_s_ = 72 mN/m with a low surface roughness (Supplementary Table 2 and Supplementary Fig. 5). However, this substrate is not transparent. Soda lime glass, onto which GPMVs spread, shows a surface tension of *γ*_s_ = 72 mN/m (Supplementary Table 2), and a low surface roughness (Supplementary Fig. 5). Apparently, borosilicate, also showing a *γ*_s_ = 72 mN/m after oxygen plasma treatment, is not smooth enough to allow GPMV spreading (Supplementary Fig. 5). We thus pursued a protocol based on a 30 nm thin evaporated SiO layer evaporated onto the borosilicate glass that remains transparent. Hot water incubation transformed the SiO layer into a SiO_x_ (1 < x < 2) layer^38^, leading to *γ*_s_ = 72 mN/m and a lowered surface roughness (Supplementary Table 2 and Supplementary Fig. 5) appropriate to successfully spread the GPMVs. With this setup, we were able to perform STED nanoscopy and reached a resolution of 60–70 nm sufficient for quantitative imaging of the Ca_V_1.3 clusters (Supplementary Fig. 3).

**Fig. 4:**
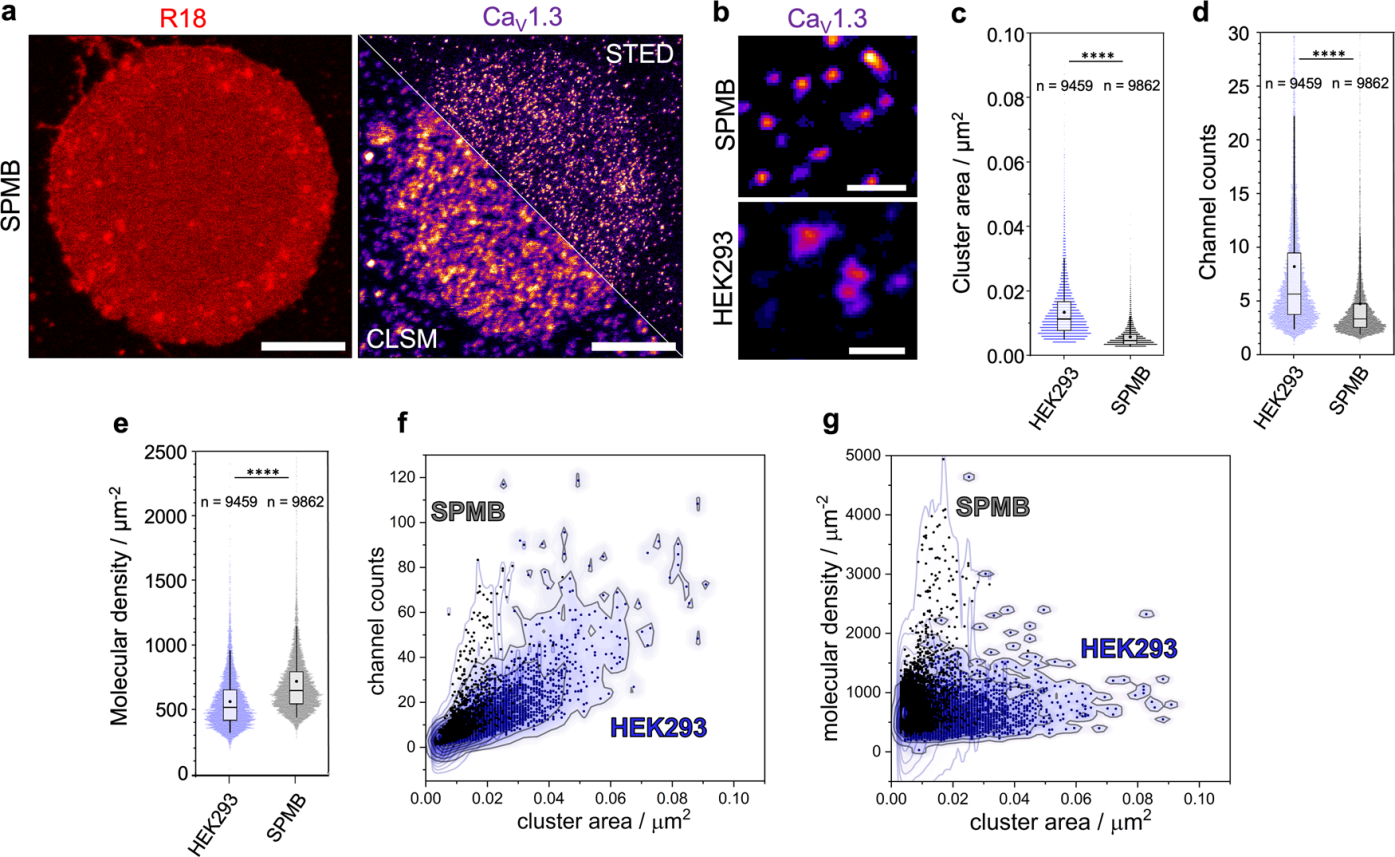
High-resolution STED images of SPMBs compared to those of living cells. **a** SPMB (R18, red) obtained upon spreading a GPMV on borosilicate glass (left image, confocal). The SPMB contains Halo-Ca_V_1.3 channel clusters (‘Fire’ LUT) (right image). The bottom part shows the confocal image, and the top part is the STED image. **b** Comparison of the appearance of the Halo-Ca_V_1.3 clusters in SPMBs and in living cells. Single Halo-Ca_V_1.3 clusters are resolved, allowing us to determine the **c** cluster area, **d** the channel counts, and **e** the molecular density (box: IQR, dot: mean, line: med.; whiskers: 5–95%) [Mann–Whitney *U*-test: ****P ≤ 0.0001]. *n* is the number of analyzed clusters. **f**, **g** Two-dimensional kernel density (2D-KDF) of Ca_V_1.3 cluster properties of cells (blue) and SPMBs (black). **f** 2D-KDF of the channel counts vs. cluster area and **g** 2D-KDF of the molecular density vs. cluster area (*n*_cell_ = 75, *n*_SPMB_ = 20). Scale bars: 10 µm (**a**); 500 nm (**b**).

With high-resolution quantitative STED nanoscopy, we resolved the Halo-Ca_V_1.3 nanoclusters within the SPMBs (Fig. 4a, Ca_V_1.3) and found a homogeneous distribution. We determined a cluster density of 1.77 ± 0.56 clusters/µm^2^. The 2.5 times larger cluster density compared to that found in living cells might indeed be attributed to a higher sensitivity and resolution resulting in the detection of smaller clusters (see below) in SPMBs. We further determined the Halo-Ca_V_1.3 cluster area in the SPMBs and determined a mean area of 0.006 ± 0.003 µm^2^ (mean ± s.d. | median: 0.005 µm^2^). This corresponds to a diameter of 86 ± 20 nm for predominantly circular clusters (Fig. 4b, top, Fig. 4c, black). Compared to the Halo-Ca_V_1.3 clusters visualized in the plasma membranes of living HEK293 cells with a mean Ca_V_1.3 cluster area of 0.014 ± 0.009 µm^2^ (median: 0.012 µm^2^, diameter: 128 ± 37 nm) (Fig. 4b, bottom, Fig. 4c, blue), the clusters in the SPMBs appear smaller. In addition to the observed smaller areas, the specimen shows lower out-of-focus light, improving the sensitivity and the signal-to-noise ratio.

This enhanced sensitivity led to more efficient detection of Halo-Ca_V_1.3 clusters that harbor fewer channel counts (5 ± 8, mean ± s.d. | median: 3.3) (Fig. 4d, black) compared to the cellular system (8 ± 9, mean ± s.d. | median: 5.6) (Fig. 4d, blue). With these data, we calculated the mean molecular density within the channel clusters in SPMBs to 709 ± 602 µm^−2^ (median: 636 µm^−2^) (Fig. 4e, black). This mean value is larger than that calculated for the mean molecular density found in cells (552 ± 217 µm^−2^, mean ± s.d. | median: 505 µm^−2^) (Fig. 4e, blue) as a result of the larger detected cluster sizes in living cells but still well below the theoretically expected value of 10,000 channels/µm^2^ given by the lateral channel dimensions of 10 nm × 10 nm^2,37^. To correlate the channel counts and molecular density to the cluster area, we plotted the two-dimensional kernel densities (Fig. 4f, g). A significant fraction of channel clusters in the plasma membrane of living cells appears not only larger in size but simultaneously displays a smaller molecular density.

**Fig. 5:**
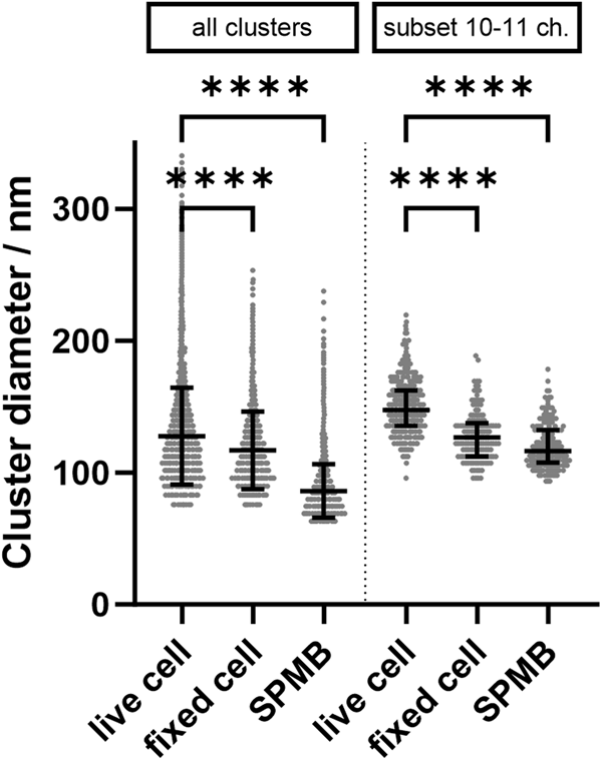
Effect of cluster mobility on the apparent cluster diameters in live cells compared to fixed cells and SPMBs. All clusters: In living cells, the mean cluster size was larger than in fixed cells and SPMBs (****p < 0.0001, ordinary one-way ANOVA, Sidak’s multiple comparison test). The sample size was *n* = 75 live cells, 15 fixed cells, and 20 SPMBs, with the corresponding clusters *n*_clusters_ = 9459, 2405, and 9862. Fixation was performed by incubating the cells with 4% paraformaldehyde + 0.1% glutaraldehyde for 10 min at room temperature. Subset: Cluster diameter of a subset of clusters containing 10–11 channel counts, a subpopulation far from the resolution limit of all systems under investigation. The sample size of this filtered dataset was *n*_clusters_ = 597, 141, and 197.

Lateral cluster mobility is expected to induce motion blurring during STED imaging. Hence, we hypothesize that the apparent smaller cluster size and, thus, larger molecular density found in SPMBs might be a result of a reduced motion blurring. To investigate this aspect, we compared the Ca_V_1.3 cluster diameters of living cells, fixed cells, and the clusters observed in SPMBs (Fig. 5). Indeed, all clusters (Fig. 5, left) appear smaller in fixed cells than in living cells as expected. In SPMBs, they appear even smaller than in fixed cells. To isolate the effect of motion blurriness from the suspected higher sensitivity in SPMBs, only a defined subset of clusters containing 10–11 channel counts was analyzed (Fig. 5, right). An object size of 10–11 channel counts is well above the resolution limit of all systems under investigation, thus excluding the resolution as a limiting factor. Indeed, cell fixation showed a much larger effect on the apparent cluster size, similar to that observed in SPMBs. These results support the notion that cluster motion contributes to the observed cluster sizes.

### Influence of β-adrenergic stimulation on the Halo-Ca_V_1.3 cluster structure in SPMBs

The possibility of imaging Ca_V_1.3 clusters in SPMBs with very high sensitivity and resolution might allow to analyze the impact of the well-known β-adrenergic receptor-selective agonist isoprenaline (or isoproterenol, ISO), previously shown to affect Ca_V_1.2 clustering using single-molecule localization microscopy^16^. We addressed the question of whether Ca_V_1.3 clusters are altered in SPMBs after cells have been treated with ISO.

**Fig. 6:**
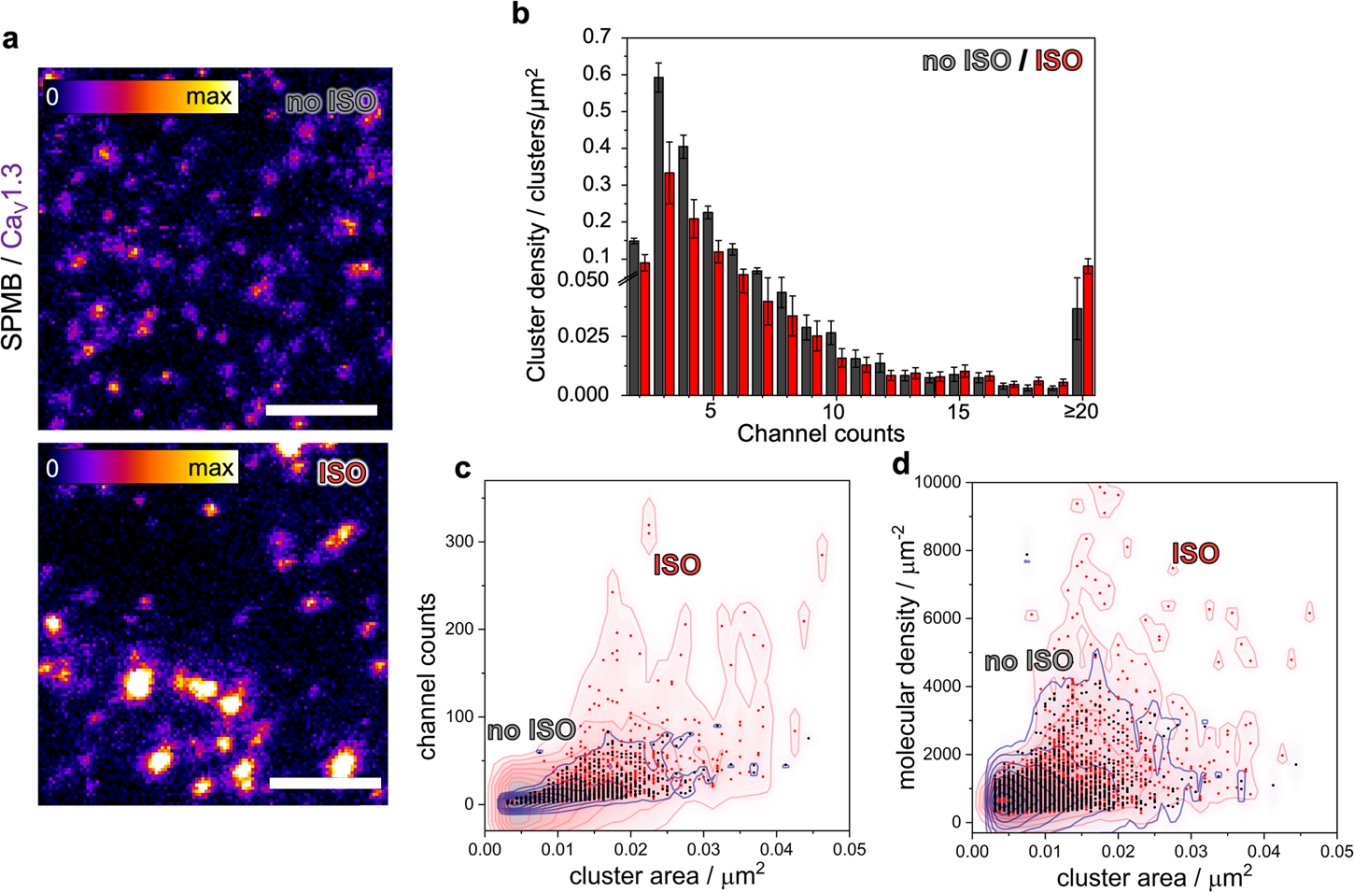
Influence of isoprenaline (ISO) on the structure of Ca_V_1.3 channel clusters in SPMBs. **a** SPMB with Halo-Ca_V_1.3 clusters (labeled with JF646-HTL, ‘Fire’ LUT, brightness scale identical for both images) without (no ISO) and with (ISO) treatment. **b** Cluster density as a function of the channel counts (mean ± s.e.m.), (untreated SPMBs, black; ISO-treated SPMBs, red). **c** Two-dimensional kernel densities (2D-KDF) of the channel counts as a function of cluster area for untreated SPMBs (black) and ISO-treated SPMBs (red). **d** 2D-KDF of the molecular density vs. cluster area for untreated (no ISO, black) and ISO-treated (ISO, red) SPMBs. Reproduced in four experiments with *n*_SPMB,no ISO_ = 20, *n*_clusters,no ISO_ = 9862; *n*_SPMB,ISO_ = 17, *n*_clusters,ISO_ = 5818. Scale bar: 1 µm (**a**).

ISO (1 μm) was applied concurrently to HaloTag labeling, preceding GPMV extraction from HEK293 cells. GPMVs were then spread as described, and STED images were taken. Visual inspection of the Halo-Ca_V_1.3 channel clusters in SPMBs reveals differences in the Ca_V_1.3 cluster density as well as the cluster size and brightness upon ISO treatment (Fig. 6a). The cluster density was reduced by about 40% from 1.77 ± 0.56 clusters/µm^2^ without ISO treatment to 1.08 ± 0.47 clusters/µm^2^ with ISO treatment, which is also reflected in an altered average inter-cluster-distance (508 versus 569 nm). The lower cluster density was accompanied by an increase in the fraction of clusters with larger channel counts at the expense of clusters with lower channel counts. Precisely, at a channel count of 13 and above, the cluster density becomes larger for the ISO-treated case compared to the untreated case (Fig. 6b). Overall, in the untreated case, cluster densities with channel counts of >13 make up 4.5% of all cluster densities, while in the ISO treated case, it is 12.3%.

This finding is mirrored by the increased cluster size heterogeneity after ISO treatment, leading to a large distribution of channel counts as a function of cluster area (Fig. 6c). To ensure that this observation is not a result of differences in the overall channel counts per unit membrane area (surface expression level, not to be confused with the channel counts within one Ca_V_1.3 channel cluster), we determined the corresponding values to be 8.6 ± 4.0 channel counts/µm^2^ for untreated SPMBs and 8.8 ± 7.0 channel counts/µm^2^ for ISO-treated SPMBs. For comparison, for living cells, we determined a value of 5.8 ± 3.8 channel counts/µm², which highlights again the overall higher sensitivity of detecting channel clusters in SPMBs.

Upon ISO treatment, the molecular density was also increased with several clusters showing a denser packing density than what was found for the untreated case. Several clusters showed molecular densities well above 5000 µm^−2^, with some even reaching molecular densities close to 10,000 µm^−2^ (Fig. 6d).

## Discussion

To date, most studies on Ca_V_1 channels still rely on indirect immunostaining strategies with the drawback of artifacts arising from the chemical fixation and an indirect, spatially distorting immunodetection^6–11^. I.e., indirect immunofluorescence combined with single-molecule localization microscopy (SMLM) has been applied to analyze the size of Ca_V_1.3 channel clusters^22^. In separate experiments, photobleaching step analysis of fluorescent protein-tagged channels was performed on diffraction-limited TIRF images using fixed cells to count individual channels within the clusters. Alternatives to immunofluorescence staining are generally based on tagging the Ca_V_1 channels with fluorescent proteins^12,13^. Here, we established a direct HaloTag high-affinity labeling strategy to allow for live-cell STED nanoscopy, which enabled us to quantify Ca_V_1.3 channel clusters concerning their size and molecular density, endosomal colocalization, and cluster regulation by the β-adrenergic agonist ISO down to the nanometer scale.

Based on this strategy, we effectively resolved individual Ca_V_1.3 channel clusters at the cell surface membrane. In accordance with other super-resolution studies of Ca_V_ channels, we predominantly found circular cluster shapes and not elongated or rectangular clusters as reported for the inner ear sensory hair cell ribbon synapse^21^ and for the largest known ion channels like the cardiac RyR2 clusters^39^. Our results are in line with the expectation that calcium channels cluster in multi-channel ensembles, leading to a spatially confined and locally tuned calcium influx for adaptable excitatory signaling^40,41^. However, the specific molecular mechanisms for Ca_V_1.3 channel clustering are still not known.

Several structural and regulatory proteins can influence cluster formation and maintenance. Confirmed interactors of Ca_V_α_1D_, the pore subunit of L-type channel clusters, include calmodulin, α-actinin, junctophilin, AKAP, and PDZ-binding proteins, all of which bind to the cytosolic C-terminal tail^42–45^. This tail has a splicing-dependent length between 180 and 694 amino acids and is not structurally rigid, thereby providing a flexible linker for dynamically clustered channels with interacting proteins. Indeed, such a clustering model is supported by previous studies, e.g. reporting on transient Ca_V_1.3 channel interactions via the C-terminus requiring Ca^2+^-bound calmodulin^42^. Interestingly, relatively small cluster areas were found for the short Ca_V_1.3 splice variant in tsA-201 cells coinciding with functional coupling^20^. Another study showed that Ca_V_1.3 clustering in neuronal cells was impaired by the deletion of the distal C-terminal PDZ domain required for scaffolding protein interaction with shank and densin^46^. Similarly, C-terminal interactions of Ca_V_2 channels were reported to mediate flexible linkage and local confinement in presynaptic nanodomains^47,48^ and for K_v_ channels to modulate clustering via a dynamic linkage to scaffolding proteins^49^. These interactions apparently not only drive and stabilize cluster formation and maintenance, respectively, but may also tune the molecular density within the clusters, which in turn changes the channel proximity to its neighbors.

We revealed subsurface cluster localization to endosomes within the cell cortex as evidenced by colocalization analysis with three endosomal Rab markers (Supplementary Table 1). Our results indicate continuous endocytosis and recycling of Ca_V_1.3 clusters at the cell surface, while clusters are structurally maintained during these processes. The estimate of 17% endosomal cluster localization at the cell surface, not taking deeper subsurface and perinuclear compartments into account, is considered an upper limit, as it cannot be ruled out that some endosomes carry a combination of Rab markers^50^. We assume that an active endosomal turnover occurs at the cell surface similar to what has been reported for the homologous channel Ca_V_1.2 in tsA-201 cells, HL-1 cells, and adult atrial cardiomyocytes^12,51,52^.

As a main result, the live-cell HaloTag-labeling strategy enabled us to count the number of channels within individual clusters using brightness-calibrated STED nanoscopy (Supplementary Fig. 3). The method is advantageous as first, it provides a defined dye-to-protein stoichiometry, leading to a predictable, linear brightness response of the target protein. Second, it features a high-affinity, irreversible ligand binding, which facilitates efficient quantitative labeling compared to less advantageous immunoreagents^53^. With this strategy, we found that most channel clusters were composed of less than 10 labeled Halo-Ca_V_1.3 molecules, which is in agreement with previously published photobleaching data^20^.

Of note, a systematic error of all cluster molecule count approaches is the total number of fluorescently labeled channels, i.e., the effective labeling efficiency (ELE). Although we did not directly determine the ELE, we can safely assume that HaloTag live-cell labeling generally achieves high ELE values of around 80%^54–56^, which would imply that the true channel number would be underestimated by a factor of 1.25. If the ELE were only 40%^57^, the factor would increase to 2.5. However, this systematic error is substantially reduced considering that an ELE of 70% was recently confirmed for DNA Origami structures^58^, which were used as a calibration standard. Taking these values into account, our potential error factor lies between 0.875 and 1.75. Within these limits, the observed molecular channel densities are still below 1250 µm^−2^. A previous study hypothesized a model of dense channel packing for Ca_V_ clusters^5^. However, as the cluster size and channel number could not be measured in the same live-cell experiments, corroborating evidence for a tight Ca_V_ clustering was missing. We were able to generate data on cluster size and channel number in parallel from the same live-cell experiment and same SPMB, allowing us to locally determine the molecular density of each individual cluster. We found molecular densities mainly in the range of 500–1000 µm^−^², which is about one order of magnitude lower than the physical limit of 10,000 µm^−2^, proposed by the dense channel packing model^5^. The size of the Ca_V_1.3 channels also ensures that the spacing of the dyes within the Halo-Ca_V_1.3 channel clusters is sufficiently large to exclude fluorophore quenching, which holds also true for brightness referencing using DNA Origami structures being appropriately far away from each other^59^.

To further push the lateral resolution to the limits, we aimed at transferring the plasma membranes of HEK293 cells to a planar support. Owing to the planarity, the lack of the cellular environment, and the reduced mobility of the membrane components, an even higher resolution was expected. Based on a previous study ^29^, we generated GPMVs derived from calmidazolium-treated HEK293 cells. We chose calmidazolium instead of the more commonly used vesiculation agents^60,61^ that are known to cross-link proteins^40,41^. Yang et al. reported that 50 μM calmidazolium did not alter the co-immunoprecipitation of Shank3 or mCherry-Ca_V_1.3 with HA-Ca_V_1.3, which was their readout for the assembly of complexes containing multiple Ca_V_1.3 channels^13^. This result suggests that calmidazolium does not interfere with channel cluster formation. Moreover, paraformaldehyde/dithiothreitol-derived GPMVs do not contain a membrane-attached F-actin network^62^. Calmidazolium-derived GPMVs partially show F-actin at the membrane interface^29^, which may contribute to a more native environment.

The HaloTag-labeled Ca_V_1.3 channels were successfully transferred from the plasma membrane of the HEK293 cells to the GPMVs. The GPMVs then spread onto the modified glass surface forming SPMB patches with a characteristic appearance^63^ that contained multiple Ca_V_1.3 channel clusters. Even though there are some reports on the spreading of GPMVs on solid support such as glass or silicon^28,29,31^, it remains still challenging as the composition of the GPMV membranes greatly varies depending on the cell line, and the protein expression profile. In general, the spreading of vesicles on support relies on parameters such as surface roughness and surface adhesion energy^64,65^. Owing to the expected reduced mobility of the clusters, fixation strategies were not required^6,46^ to obtain high-resolution images with minimized motion blurring and without apparent photodamage.

However, even though we could indeed detect channel clusters in the SPMBs with only three channel counts, we found a molecular density in the SPMBs that was still in the main range of 500–1000 µm^−2^. Compared to the data obtained for living cells, the mean value (709 µm^−2^) was about 30% larger but still much lower than the theoretical limit^5^.

Owing to the very high resolution of the clusters in SPMBs, the system appeared to be suited to investigate the influence of the sympathomimetic compound ISO on the calcium channel clusters, based on, at least in part, a Rad-dependent mechanism leading to increased Ca^2+^ influx^66^. ISO treatment has been shown to augment Ca_V_1.2 clustering in tsA-201 cells and isolated mouse cardiomyocytes^52^. We found that after ISO treatment of HEK293 cells, from which the GPMVs were derived, the overall cluster density in the resulting SPMBs was reduced by ~40% at the expense of a larger heterogeneity of the clusters with an increased number of larger and brighter clusters. ISO treatment generated a fraction of channel clusters observed in the SPMBs with counted channel densities over 5000 µm^−2^. A few clusters even reached the 10,000 µm^−2^ limit. To date, no data on Ca_V_1.3 cluster densities upon ISO treatment were available. However, ref. ^16^ reported on Ca_V_1.2 cluster sizes as a function of ISO treatment. They imaged fixed Ca_V_β_2a_-paGFP transduced cardiomyocytes in TIRF mode with 150 nm penetration depth, on a super-resolution-ground state depletion (GSD) microscope and found an increased number of Ca_V_1.2 channels per cluster upon ISO stimulation (mean number of Ca_V_β_2a_-paGFP molecules per cluster: 6.62 ± 0.16 (−ISO), 8.75 ± 0.22 (+ISO)). They attributed this finding to the closer proximity of single channels in super-clusters, which enhances channel cooperativity that amplifies Ca^2+^ influx. Of note, HEK293 cells differ significantly from the β-adrenergic receptor reservoir in cardiomyocytes, which express at least two functional β_1_ and β_2_ isoforms with distinct subcellular functions^67^. However, even though it is conceivable that ISO stimulates the coalescence of clusters leading to super-clusters, in line with the β-adrenergic stimulation generally increasing channel function^52^, the mechanism of super-cluster formation is as yet unknown.

To conclude, HaloTag-based live-cell nanoscopy of L-type Ca_V_1.3 channels is a valuable stoichiometric tool to decipher the nanoscale arrangement of these channel clusters. The strategy has the potential to be applied also to other channel types forming ion channel clusters. In conjunction with planar SPMBs, allowing to apply highly complementary methods such as atomic force microscopy, molecule tracking, or MINFLUX nanoscopy^68^, this approach may open up new avenues to understand calcium channel cluster formation, maintenance, turnover, and local functions in distinct cell types and tissues.

## Methods

### Plasmids

Ca_V_1.3 human cDNA (accession number NM_001128840.2) was synthesized including an N-terminal ‘GGS’ linker. This cDNA was assembled into a vector encoding N-terminal fusion to the HaloTag (Promega G7721) using restriction cloning, yielding the Halo-Ca_V_1.3 plasmid. To alternatively generate an N-terminal mEGFP fusion, HaloTag was exchanged by restriction cloning to yield the GFP-Ca_V_1.3 plasmid. Lastly, an alternative Ca_V_1.3 cDNA sequence was synthesized containing the insertion of a ‘GGS’-flanked HA-tag into domain II loop S5S6 of Ca_V_1.3, resulting in the Ca_V_1.3-HA plasmid^69^.

GFP-Rab11a^70^ was a gift from Richard Pagano (Addgene plasmid # 12674). GFP-Rab4a^71^ was a gift from Marci Scidmore (Addgene plasmid # 49434). GFP-Rab5a was expressed by baculoviral transduction using CellLight™ Early Endosomes GFP reagent (Invitrogen C10586).

### Cell culture and transfection

A HEK293 cell line with constitutive expression of Ca_V_ subunits β_3_ and α_2_δ_1_ and inducible expression of α_1D_ (Charles River Laboratories, CT6232) was used throughout this study and cultured in DMEM/F12 medium containing selection antibiotics and 0.6 µM isradipine. For imaging experiments, cells were seeded on fibronectin-coated glass-bottom imaging dishes (ibidi 81158) in a growth medium lacking selection antibiotics. For SPMB experiments, cells were seeded likewise on six-well culture plates. Transfection was performed using Lipofectamine 3000 reagent (Thermo Fisher L3000008) mixed with Halo-Ca_V_1.3 plasmid (0.6 µg per imaging dish, 2 µg per well for six-well plates) in OptiMEM. For colocalization studies, either 0.3 µg GFP-Rab11a or 0.6 µg GFP-Rab4a was added to the transfection mix, or GFP-Rab5a reagent (4 × 10^6^ viral particles) was added besides lipofection. A washing step was performed 3 h after transfection using a fresh culture medium. For control conditions, Ca_V_1.3^WT^ (α_1D_) was induced by adding 1 µg/ml tetracycline to the culture medium in parallel to transfections. Microscopy experiments were carried out 2 d after transfection.

### Cell labeling for microscopy

For each HaloTag imaging experiment, a labeling solution containing 200 nM JF646-HTL (Promega GA1121) in phenol red-free culture medium was freshly prepared. Live-cell labeling was performed by incubation of HEK293 cells in labeling solution for 20 min at 37 °C, followed by optional co-labeling by Cellmask PM (Thermo Fisher C10046) or 0.2 µm R18 membrane dye (Thermo Fisher O246) or Cellmask Actin (Thermo Fisher A57234) according to the manufacturer’s instructions. After labeling, a wash-out step was performed by incubation with fresh culture medium for 1 h at 37 °C. Afterward, cells were washed thrice and imaged in a live-cell imaging solution (Thermo Fisher A14291DJ). For the treatment with isoprenaline (ISO), 1 µM ISO was added to the labeling solution and GPMV formation was induced as described below.

For immunofluorescence, cells were first labeled with JF646-HTL, as described above. Afterward, cells were fixed for 10 min with 4% paraformaldehyde diluted in phosphate-buffered saline (PBS), then blocked and permeabilized for 1 h using 10% bovine calf serum and 0.1% Triton X-100 in PBS and then incubated with the primary antibody in blocking buffer overnight at 4 °C. This was followed by a secondary antibody incubation in a blocking buffer for 90 min at room temperature. The following antibodies were used: Anti-Ca_V_1.3 polyclonal rabbit antibody (Alomone Labs ACC-005, dilution 1:100) and anti-Ca_V_1.3 mouse monoclonal antibody (Aviva Biosystems OASE00151, dilution 1:200) both raised against AA 859-875 of rat Ca_V_1.3.

### GPMV formation

To induce GPMV formation, PBS was exchanged against 1 mL GPMV buffer (10 mM HEPES, 150 mM NaCl, 2 mM CaCl_2_, pH 7.4). 1 mL of calmidazolium was added to a cell culture of ~70% cell confluency, reaching a final concentration of 10 µm. Then, cells were incubated at 37 °C for 3 h. The GPMV solution was gently extracted with a cutoff glass pipette closely above the cell layer but avoiding contact to reduce friction forces. To prevent contact with any plastics, GPMVs were transferred to flat top glass 50 × 10.5 mm^2^ containers, as some GPMVs undesirably might spread on plastics. Before substrate activation, the GPMV solution was diluted 1:9.

### SPMB formation

In the case of upright STED imaging, soda lime glass (microscope slides DIN ISO 8037-1, Epredia) was activated by an oxygen plasma [*p*(O_2_) = 0.2 mbar, *E*_output_ = 13.7 J, *t* = 3 s, *d* = 9.5 cm] (Plasma cleaner Zepto, Diener electronic GmbH + Co.KG)^29^. In the case of inverse STED imaging, borosilicate glass was coated with 30 nm of SiO (UNIVEX400, Leybold)^38^. Hot water incubation was performed at 80 °C for 30 min. GPMVs were spread immediately after incubation to form SPMBs^29^.

### Confocal and STED imaging

Confocal and STED imaging was performed using the following microscopes: Abberior Expert Line STED 775 QUAD Scan, inverted setup, NA 1.4 oil immersion objective lens (UPlanSApo 100x, Olympus), pulsed excitation lasers at wavelengths 640/591/485 nm, pulsed STED laser at wavelength 775 nm, detection by avalanche photodiodes (SPCM-AQRH, Excelitas Technologies Corp.). STEDYCON upright setup, NA 1.1 water immersion objective lens (LUMFLN60X-W, Olympus), pulsed excitation lasers at wavelengths 646/561/488 nm, pulsed STED laser at wavelength 775 nm.

Acquisition parameters were optimized, balancing spatial resolution, temporal resolution, and sensitivity. Settings for Abberior Expert Line (inverted setup): 30% excitation laser power at 640 nm, 12% STED laser power, pixel size 30 nm (live-cell)/25 nm (SPMBs), pixel dwell time 36 µs (live-cell)/64 µs (SPMBs), time gating window 0.5–6 ns. Settings for STEDYCON (upright setup): 10% excitation laser power at 488 nm, 3/10% excitation laser power at 488 nm (SPMB/close-up), 100% excitation laser power at 640 nm, 70/99% STED laser at 775 nm (SPMB/close-up), pixel size 30 nm, pixel dwell time 30 µs/250 µs (SPMB/close-up), time gating window 1–7 ns, 1×/25× line accumulation (SPMB/close-up).

### Brightness referencing

DNA Origami reference structures containing 23 ± 3 and 7 ± 1 JF646 dye binding sites were ordered from GATTAquant. The samples were immobilized on the surface of ibidi glass-bottom imaging dishes by BSA-biotin coating and then immersed in an imaging buffer corresponding to live-cell or SPMB experiments. Brightness referencing was performed by applying the same quantitative nanoscopy workflow as for Halo-Ca_V_1.3 cluster samples. The resulting distribution of single particle brightness was used to determine a single dye brightness value for each experimental setup, which was used for molecular counting of channel molecules.

### Image processing and analysis

Shown images represent raw data, in some cases filtered by a “Gaussian Blur” and “Subtract Background” commands for display purposes. For STED image segmentation, an FFT bandpass filter (2.5-20 px) was applied to remove high-frequency noise and an unstructured background. Candidate signal spots were identified in the filtered image by maxima detection and peak expansion to half-maximal intensity (FWHM) using the ImageJ plugins *FindFoci*^72^ and *Interactive H-Watershed* (https://github.com/mpicbg-scicomp/Interactive-H-Watershed/). Resulting candidate regions of interest (ROI) were discarded if containing less than 5 px or a mean intensity less than 50% above the background. All remaining ROIs representing specific signals were used for area and brightness measurements on raw image data. For brightness measurements, the local background was subtracted for each ROI using the mean brightness in a ring-like ROI obtained by differential ROI enlargement by 4 vs 2 px.

Endosomal images were binarized using bandpass filtering (4–30 px) followed by automated thresholding (Rab4/5 ‘Triangle’, Rab11 “Li” (live-cell), Rab4/5/11 “mean of background” (SPMB close-ups). Colocalization was determined from binary maps as the fraction of Ca_V_1.3 cluster spots overlapping with endosomal spots. To correct for nonspecific colocalization, the area fraction of each endosomal signal was used to approximate the random overlap probability and was subtracted from the initial colocalizing fraction to yield the specific colocalization values as reported in the text and figures. Quantification of pixel distances was performed by pixel-wise enlargement of ROIs until overlap was reached. Nearest neighbor distance (NND) analysis for clusters was performed from center to center.

### Statistics and reproducibility

Image analysis data were visualized and statistically analyzed using GraphPad Prism version 9.5.0/ 10.1.2, and OriginPro 2020. Data obtained from each experiment were expressed as the mean ± s.e.m. or mean ± s.d. as indicated. Median values were reported for skewed data distributions. Histograms and scatter plots were used rather than bar graphs to reflect the full data distributions. For statistical testing, the significance between the two groups was determined by two-tailed tests, as stated in the figure captions. Significance levels (****p ≤ 0.0001) and sample sizes were described in each figure legend.

### Reporting summary

Further information on research design is available in the Nature Portfolio Reporting Summary linked to this article.

### Supplementary information

Supplemental Information

Description of Additional Supplementary Materials

Supplementary Data 1

Reporting summary

## Data Availability

All data needed to evaluate the conclusions in the paper are present in the paper and/or the Supplementary Material. The source data behind the graphs in the paper are found in Supplementary Data 1. Additional data related to this paper may be requested from the authors.

## References

[1] Pitt GS, Matsui M, Cao CK (2021). Voltage-gated calcium channels in nonexcitable tissues. Annu. Rev. Physiol..

[2] Zamponi GW, Striessnig J, Koschak A, Dolphin AC (2015). The physiology, pathology, and pharmacology of voltage-gated calcium channels and their future therapeutic potential. Pharmacol. Rev..

[3] Dixon RE (2021). Nanoscale organization, regulation, and dynamic reorganization of cardiac calcium channels. Front. Physiol..

[4] Zuccotti A (2011). Structural and functional differences between L-type calcium channels: crucial issues for future selective targeting. Trends Pharmacol. Sci..

[5] Sato D (2019). A stochastic model of ion channel cluster formation in the plasma membrane. J. Gen. Physiol..

[6] Ichikawa T (2022). Chemical fixation creates nanoscale clusters on the cell surface by aggregating membrane proteins. Commun. Biol..

[7] Huebinger J, Spindler J, Holl KJ, Koos B (2018). Quantification of protein mobility and associated reshuffling of cytoplasm during chemical fixation. Sci. Rep..

[8] Sograte-Idrissi S (2020). Circumvention of common labelling artefacts using secondary nanobodies. Nanoscale.

[9] Schnell U, Dijk F, Sjollema KA, Giepmans BN (2012). Immunolabeling artifacts and the need for live-cell imaging. Nat. Methods.

[10] Irgen-Gioro, S., Yoshida, S., Walling, V. & Chong, S. Fixation can change the appearance of phase separation in living cells. *Elife*10.7554/eLife.79903 (2022). 10.7554/eLife.79903 PMC9817179 36444977

[11] Richter KN (2018). Glyoxal as an alternative fixative to formaldehyde in immunostaining and super-resolution microscopy. EMBO J..

[12] Conrad R, Kortzak D, Guzman GA, Miranda-Laferte E, Hidalgo P (2021). Ca_V_b controls the endocytic turnover of Ca_V_1.2 L‐type calcium channel. Traffic.

[13] Yang Q (2023). Clustering of Ca_V_1.3 L-type calcium channels by Shank3. J. Neurochem..

[14] De La Mata A (2019). BIN1 induces the formation of T-tubules and adult-like Ca^2+^ release units in developing cardiomyocytes. Stem Cells.

[15] Dixon, R. E. et al. Graded Ca^2+^/calmodulin-dependent coupling of voltage-gated Ca_V_1.2 channels. *Elife*10.7554/eLife.05608 (2015). 10.7554/eLife.05608 PMC4360655 25714924

[16] Ito DW (2019). b-adrenergic-mediated dynamic augmentation of sarcolemmal Ca_V_1.2 clustering and co-operativity in ventricular myocytes. J. Physiol..

[17] Striessnig J, Pinggera A, Kaur G, Bock G, Tuluc P (2014). L-type Ca^2+^ channels in heart and brain. Wiley Interdiscip. Rev. Membr. Transp. Signal.

[18] Brandenburg S (2016). Axial tubule junctions control rapid calcium signaling in atria. J. Clin. Invest..

[19] Zhang Z (2005). Functional roles of Ca_V_1.3 (alpha1D) calcium channels in atria: insights gained from gene-targeted null mutant mice. Circulation.

[20] Moreno, C. M. et al. Ca^2+^ entry into neurons is facilitated by cooperative gating of clustered Ca_V_1.3 channels. *Elife* **5**, e15744 (2016). 10.7554/eLife.15744 PMC4869912 27187148

[21] Neef J (2018). Quantitative optical nanophysiology of Ca^2+^ signaling at inner hair cell active zones. Nat. Commun..

[22] Dixon RE, Navedo MF, Binder MD, Santana LF (2022). Mechanisms and physiological implications of cooperative gating of clustered ion channels. Physiol. Rev..

[23] Schlegel J (2023). A multiparametric and high-throughput platform for host-virus binding screens. Nano Lett..

[24] Sezgin E (2022). Giant plasma membrane vesicles to study plasma membrane structure and dynamics. Biochim. Biophys. Acta Biomembr..

[25] Shelby SA, Castello-Serrano I, Wisser KC, Levental I, Veatch SL (2023). Membrane phase separation drives responsive assembly of receptor signaling domains. Nat. Chem. Biol..

[26] Florentsen CD (2021). Annexin A4 trimers are recruited by high membrane curvatures in giant plasma membrane vesicles. Soft Matter.

[27] Gerstle Z, Desai R, Veatch SL (2018). Giant plasma membrane vesicles: an experimental tool for probing the effects of drugs and other conditions on membrane domain stability. Methods Enzymol.

[28] Chiang PC, Tanady K, Huang LT, Chao L (2017). Rupturing giant plasma membrane vesicles to form micron-sized supported cell plasma membranes with native transmembrane proteins. Sci. Rep..

[29] Teiwes NK (2021). Pore-spanning plasma membranes derived from giant plasma membrane vesicles. ACS Appl. Mater. Interfaces.

[30] Sundaram, R. V. K. et al. Native planar asymmetric suspended membrane for single-molecule investigations: plasma membrane on a chip. *Small* **18**, e2205567 (2022). 10.1002/smll.202205567 36328714

[31] Sezgin, E., Carugo, D., Levental, I., Stride, E. & Eggeling, C. Creating supported plasma membrane bilayers using acoustic pressure. *Membranes*10.3390/membranes10020030 (2020). 10.3390/membranes10020030 PMC7074417 32085393

[32] Zhang H (2005). Association of Ca_V_1.3 L-type calcium channels with Shank. J. Neurosci..

[33] Grimm JB (2015). A general method to improve fluorophores for live-cell and single-molecule microscopy. Nat. Methods.

[34] Lachmanovich E (2003). Co-localization analysis of complex formation among membrane proteins by computerized fluorescence microscopy: application to immunofluorescence co-patching studies. J. Microsc..

[35] Schmied JJ (2012). Fluorescence and super-resolution standards based on DNA origami. Nat. Methods.

[36] Hummert J, Tashev SA, Herten DP (2021). An update on molecular counting in fluorescence microscopy. Int. J. Biochem. Cell Biol..

[37] Yao X, Gao S, Yan N (2022). Structural basis for pore blockade of human voltage-gated calcium channel Ca_V_1.3 by motion sickness drug cinnarizine. Cell Res..

[38] Teske N (2017). Continuous pore-spanning lipid bilayers on silicon oxide-coated porous substrates. Langmuir.

[39] Baddeley D (2009). Optical single-channel resolution imaging of the ryanodine receptor distribution in rat cardiac myocytes. Proc. Natl Acad. Sci. USA.

[40] Lang T, Rizzoli SO (2010). Membrane protein clusters at nanoscale resolution: more than pretty pictures. Physiology.

[41] Meyer AC (2009). Tuning of synapse number, structure and function in the cochlea. Nat. Neurosci..

[42] Kuzmenkina E, Novikova E, Jangsangthong W, Matthes J, Herzig S (2019). Single-channel resolution of the interaction between C-terminal Ca_V_1.3 isoforms and calmodulin. Biophys. J..

[43] Choi S, Vivas O, Baudot M, Moreno CM (2022). Aging alters the formation and functionality of signaling microdomains between L-type calcium channels and b2-adrenergic receptors in cardiac pacemaker cells. Front. Physiol..

[44] Yang ZF (2022). Structures of the junctophilin/voltage-gated calcium channel interface reveal hot spot for cardiomyopathy mutations. Proc. Natl Acad. Sci. USA.

[45] Turner M (2020). a-Actinin-1 promotes activity of the L-type Ca^2+^ channel Ca_V_1.2. EMBO J.

[46] Stanika R (2016). Splice variants of the Ca_V_1.3 L-type calcium channel regulate dendritic spine morphology. Sci. Rep..

[47] Heck J (2019). Transient confinement of Ca_V_2.1 Ca^2+^-channel splice variants shapes synaptic short-term plasticity. Neuron.

[48] Gardezi SR, Nath AR, Li Q, Stanley EF (2016). Characterization of a synaptic vesicle binding motif on the distal Ca_V_2.2 channel C-terminal. Front. Cell Neurosci..

[49] Lewin L (2020). Molecular and cellular correlates in Kv channel clustering: entropy-based regulation of cluster ion channel density. Sci. Rep..

[50] Sonnichsen B, De Renzis S, Nielsen E, Rietdorf J, Zerial M (2000). Distinct membrane domains on endosomes in the recycling pathway visualized by multicolor imaging of Rab4, Rab5, and Rab11. J. Cell Biol..

[51] Ghosh D (2018). Dynamic L-type Ca_V_1.2 channel trafficking facilitates Ca_V_1.2 clustering and cooperative gating. Biochim. Biophys. Acta Mol. Cell Res..

[52] Del Villar, S. G. et al. β-Adrenergic control of sarcolemmal Ca_V_1.2 abundance by small GTPase Rab proteins. *Proc. Natl Acad. Sci. USA*10.1073/pnas.2017937118 (2021). 10.1073/pnas.2017937118 PMC7896340 33558236

[53] Encell LP (2012). Development of a dehalogenase-based protein fusion tag capable of rapid, selective and covalent attachment to customizable ligands. Curr. Chem. Genomics.

[54] Dickinson DJ, Schwager F, Pintard L, Gotta M, Goldstein B (2017). A single-cell biochemistry approach reveals PAR complex dynamics during cell polarization. Dev. Cell.

[55] Lepore A (2019). Quantification of very low-abundant proteins in bacteria using the HaloTag and epi-fluorescence microscopy. Sci. Rep..

[56] Liu AA (2016). Simultaneous visualization of parental and progeny viruses by a capsid-specific HaloTag labeling strategy. ACS Nano.

[57] Thevathasan JV (2019). Nuclear pores as versatile reference standards for quantitative superresolution microscopy. Nat. Methods.

[58] Hummert J (2021). Photobleaching step analysis for robust determination of protein complex stoichiometries. Mol. Biol. Cell.

[59] Schroder T, Scheible MB, Steiner F, Vogelsang J, Tinnefeld P (2019). Interchromophoric interactions determine the maximum brightness density in DNA Origami structures. Nano Lett..

[60] Levental KR, Levental I (2015). Isolation of giant plasma membrane vesicles for evaluation of plasma membrane structure and protein partitioning. Methods Mol. Biol..

[61] Baumgart T (2007). Large-scale fluid/fluid phase separation of proteins and lipids in giant plasma membrane vesicles. Proc. Natl Acad. Sci. USA.

[62] Schneider F (2017). Diffusion of lipids and GPI-anchored proteins in actin-free plasma membrane vesicles measured by STED-FCS. Mol. Biol. Cell.

[63] Hamai C, Cremer PS, Musser SM (2007). Single giant vesicle rupture events reveal multiple mechanisms of glass-supported bilayer formation. Biophys. J..

[64] Raedler J, Strey H, Sackmann E (2002). Phenomenology and kinetics of lipid bilayer spreading on hydrophilic surfaces. Langmuir.

[65] Cremer PS, Boxer SG (1999). Formation and spreading of lipid bilayers on planar glass supports. J. Phys. Chem. B.

[66] Papa, A. et al. A membrane-associated phosphoswitch in Rad controls adrenergic regulation of cardiac calcium channels. *J. Clin. Invest.*10.1172/JCI176943 (2024). 10.1172/JCI176943 PMC10904049 38227371

[67] Nikolaev VO (2010). Beta2-adrenergic receptor redistribution in heart failure changes cAMP compartmentation. Science.

[68] Schmidt R (2021). MINFLUX nanometer-scale 3D imaging and microsecond-range tracking on a common fluorescence microscope. Nat. Commun..

[69] Jenkins MA (2010). Ca^2+^-dependent facilitation of Ca_V_1.3 Ca^2+^ channels by densin and Ca^2+^/calmodulin-dependent protein kinase II. J. Neurosci..

[70] Choudhury A (2002). Rab proteins mediate Golgi transport of caveola-internalized glycosphingolipids and correct lipid trafficking in Niemann-Pick C cells. J. Clin. Invest..

[71] Rzomp KA, Scholtes LD, Briggs BJ, Whittaker GR, Scidmore MA (2003). Rab GTPases are recruited to chlamydial inclusions in both a species-dependent and species-independent manner. Infect. Immun..

[72] Herbert AD, Carr AM, Hoffmann E (2014). FindFoci: a focus detection algorithm with automated parameter training that closely matches human assignments, reduces human inconsistencies and increases speed of analysis. PLoS ONE.

