# Supplementary material for: CaV1.3 channel clusters characterized by live-cell and isolated plasma membrane nanoscopy
Source: Commun Biol. 2024 May 23;7:620. doi: 10.1038/s42003-024-06313-3 (PMC11116533; doi:10.1038/s42003-024-06313-3)
Supplement: Supplementary file 1 — Supplemental Information [file 42003_2024_6313_MOESM1_ESM.pdf]

Supplementary Information

Validation of Halo-Ca$_V$1.3 protein expression and imaging

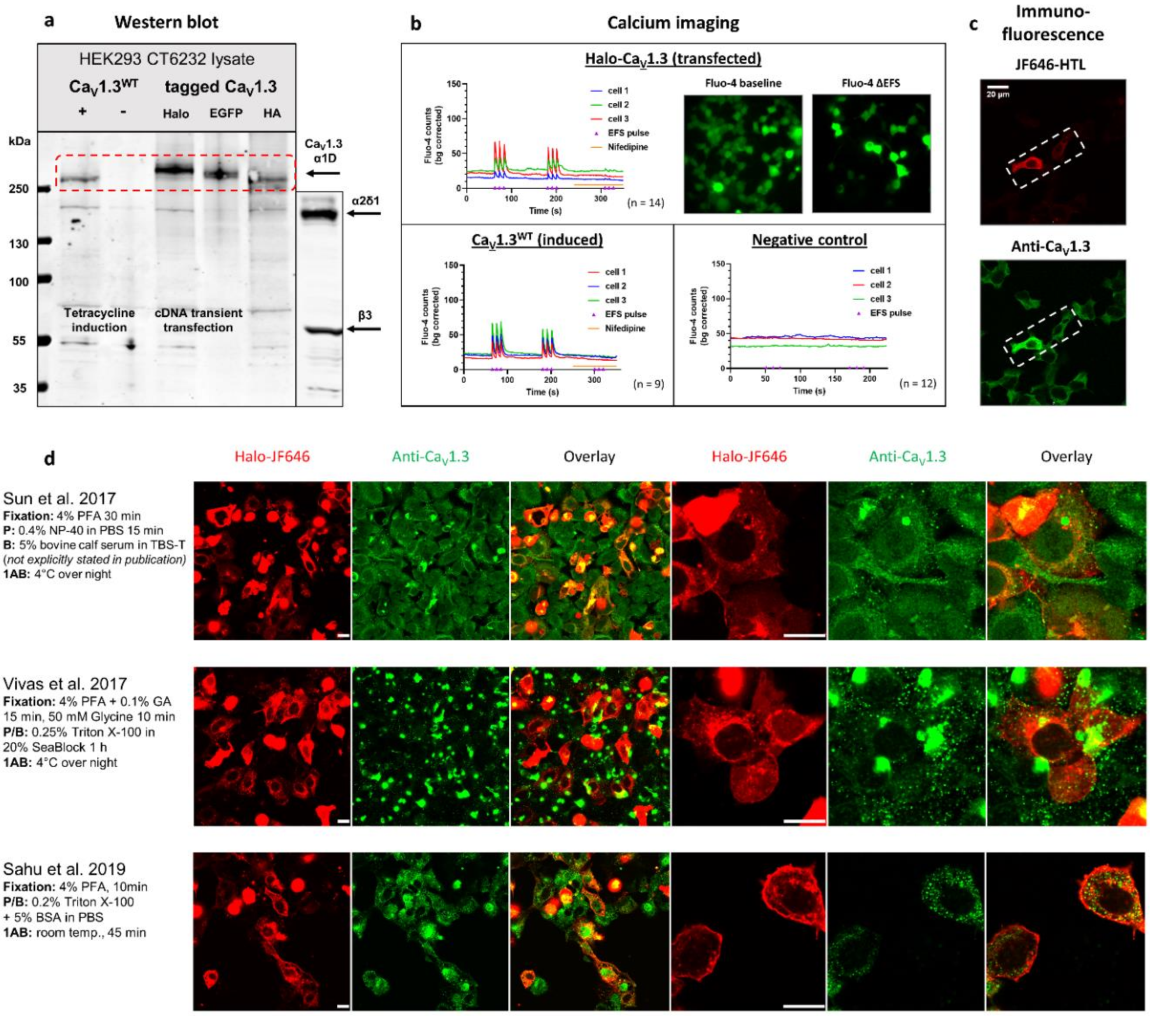

**Supplementary Fig. 1 | Validation of Halo-Ca$_V$1.3 protein expression and imaging. a,** Western blot showing the detection of full-length Ca$_V$1.3 including accessory channel subunits in protein lysates of transiently transfected HEK293 cells as indicated for each column. **b,** Calcium imaging in Fluo-4 loaded HEK293 cells confirmed functional surface expression of Halo-Ca$_V$1.3. Calcium signals were evoked by electric field stimulation (30 V, 0.1 Hz) and specifically inhibited by the application of 10 µM nifedipine. **c,** Confocal image of HEK293 cells labeled with the Halo-Ca$_V$1.3 ligand JF646-HTL and mouse anti-Ca$_V$1.3 immunofluorescence showing that transfected cells at high expression levels are detected by both labeling strategies (bright cell in the marked region). However, a higher level of the background was observed by antibody labeling, which precluded the detection of clustered transfected cells with low-intensity signals (dark cell in the marked region compared to surrounding cells) in contrast to JF646-HTL labeling. **d,** Halo-Ca$_V$1.3 labeling by indirect immunofluorescence (IF) using different published protocols[1-3] with a literature-established rabbit anti-Ca$_V$1.3 antibody (Alomone ACC-005). The IF stainings were performed on HEK293 cells expressing Halo-Ca$_V$1.3, which was labeled by JF646-HTL as a positive control. Each tested protocol resulted in major nonspecific antibody binding, as evident by variable background staining patterns in non-transfected cells and an overall lack of correlation with Halo-JF646 signals. Similar results were obtained in multiple independent experiments using different antibody production batches and concentrations. We conclude that faithful Ca$_V$1.3 staining could not be reproduced with the tested antibody. ‘P’ = Permeabilization step; ‘B’ = Blocking step; ‘1AB‘ = Primary antibody incubation conditions in the respective blocking buffer. Scale bars: 20 µm.

Colocalization of endosomal markers and $Ca_V1.3$ clusters

Supplementary Table 1 | Colocalization of three different RAB-markers with $Ca_V1.3$ clusters. Errors are given as s.e.m.

	$Ca_V1.3$-coloc. / %	*N*	*n*
**RAB4a**	8.4 ± 1.1	3	17
**RAB5a**	3.8 ± 0.7	3	30
**RAB11a**	5.0 ± 1.6	3	28
**Sum**	17.2		

N = number of experiments, *n* = number of cells.

Brightness referencing and photobleaching step analysis

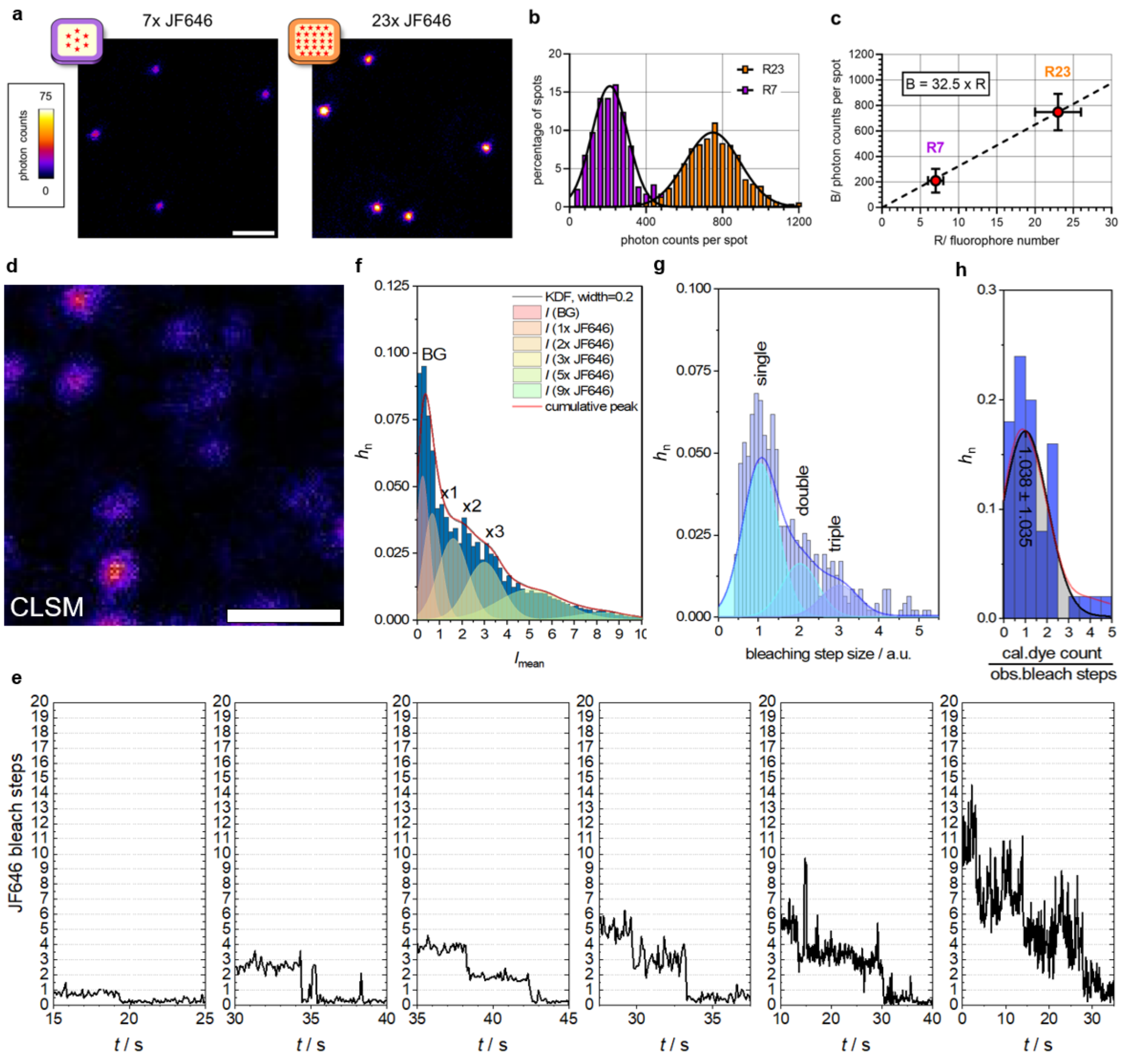

Supplementary Fig. 2 | Brightness referencing for molecular counting of JF646, validated by photobleaching step analysis. a, DNA Origami linked to 7 or 23 JF646 dye molecules were immobilized on coverslips and STED images were taken. The imaging was performed under equal conditions as for Halo-Cav1.3 cluster analysis. **b,** Distribution of integrated photon counts for all detected spots. **c,** Gaussian fit-derived mean values to determine a linear conversion factor of spot brightness to fluorophore number. Given mean signal maxima of 1.1 MHz for spots containing 23 fluorophores, APD detector saturation was negligible at only 3%, leading to a linear dependency of molecule counts and brightness. The shown calibration applies to SPMB data on borosilicate glass; a corresponding analysis was performed using live cell and SPMB imaging acquisition parameters. To prove whether the resulting brightness was related to the dye molecule counts, single-molecule photobleaching was performed on Cav1.3 clusters. To determine the Origami DNA-calibrated brightness of the clusters, they were imaged once with SPMB imaging conditions before single-molecule photobleaching was performed. ROIs were defined in the STED images and used for the CLSM images (CLSM image before photobleaching is shown in **d**) to ensure precise cluster cropping. Bleaching of 72 clusters (examples depicted in **e**) was performed for 50 s at 10 fps. **f,** Corresponding intensity histogram (n_{frame} = 36000) (h_n: relative frequency, with a kernel density function, KDF shown as a red line). Using Gaussian unmixing expectancy, equidistantly distributed values were found. A per-step size of 0.9 a.u was determined. **g,** Histogram of the bleaching step sizes (Gaussian unmixing: transparent, KDF: blue line). **e,** Rescaling the intensity to bleach steps, single tracks show the number of dyes bleached. **h,** Comparing the bleach step calibration and the Origami DNA brightness calibration of the same 72 Cav1.3 clusters, a ratio of 1.038 ± 1.035 molecule counts per observed bleached dye (mean ± s.d.) was calculated, proving that both calibrations result, on average, in the same number of dye molecules (Gaussian fit: black line + transparent, KDF: red line). Scale bars **a**, **d**: 1 µm.

Resolution of the STED microscope and impact on the cluster detection

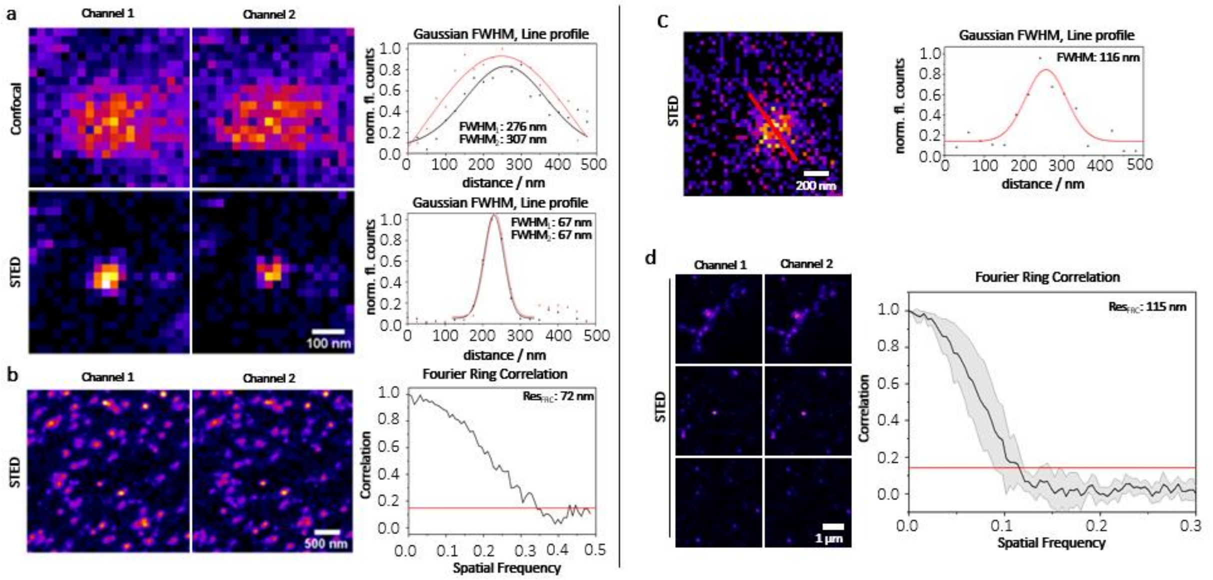

Supplementary Fig. 3 | Inverse and upright STED image resolution measured by line profiles and Fourier ring correlation. a, b, Inverse STED: The images depict SPMBs on borosilicate glass containing Halo-Ca$_V$1.3 clusters, recorded as two interleaved channels with acquisition settings as used in experiments. **a,** Line profile analysis and **b,** Fourier ring correlation of both channels show that a lateral resolution of around 70 nm was reached. **c, d,** Upright STED: SPMBs on borosilicate glass containing Halo-Ca$_V$1.3 clusters were imaged and analyzed to determine the resolution by line scans and Fourier ring correlation. **c,** Line profile analysis, and **d,** Fourier ring correlation. Both methods provide a lateral resolution of 115 nm (mean, s.d.: lower: 100 nm, upper: 117 nm).

Owing to the differences in resolution, the spot density of Halo-Ca$_V$1.3 clusters in SPMBs on borosilicate glass, detected by inverse STED microscopy, was 1.77 ± 0.56 clusters/µm^2, which is about 25 times larger than the detected mean spot density of SPMBs on soda lime glass (0.07 ± 0.13 spots/µm^2) reflecting the superior sensitivity and resolution of the inverse STED microscope. The spot density found for HEK293 cells was 0.69 ± 0.06 clusters/µm^2 being less than that found in SPMBs on borosilicate glass even though the same microscope was used, and we assume a one-to-one transfer. This finding might be a result of the higher signal-to-noise ratio leading to improved separation of small clusters in SPMBs. Indeed, 94% of the detected Halo-Ca$_V$1.3 clusters in the SPMBs on borosilicate glass were smaller than 10 channel counts per cluster, whereas in HEK293 cells only 79% were found.

Determination of the buffer exposed side of SPMBs

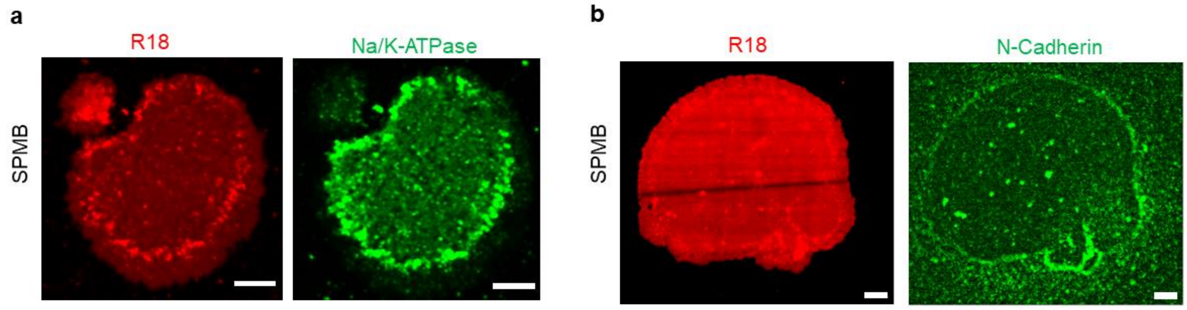

Supplementary Fig. 4 | Membrane side specific antibody labeling for elucidating GPMV spreading mechanism. a,b, R18 doped SPMB patches (red) derived from spreading GPMVs on an oxygen plasma-activated soda-lime glass. Two differently labeled antibodies (green) were applied to detect proteins specific to the intracellular or extracellular side of the membrane. After passivating the surface with a 0.2 % bovine serum albumin solution, the surface was treated with **a,** the monoclonal mouse primary antibody anti-Na/K-ATPase (1:200, Alomone, ANP-001), which binds to the intracellular side of the protein or **b**, with the mouse anti-N-cadherin (1:300, Merck, C3865) binding to the extracellular domains of the protein. After rinsing, the surface was incubated with an anti-mouse 2nd antibody labeled with Alexa Fluor488 (Invitrogen, A32723). The antibody directed to the intracellular side of the GPMV binds strongly to the SPMB, whereas the antibody directed to the extracellular side does not show specific binding to the SPMB indicating that the extracellular side of the GPMV-derived membranes faces the substrate. Scale bars: 5 µm.

Characterization of the surfaces used to spread GPMVs

Hydrophilic surfaces are required to spread GPMVs onto a substrate. A substrate surface tension, determined by contact angle measurements, of $\gamma_s \geq 72$ mN/m appeared to be mandatory for spreading (Supplementary Table 2). However, although borosilicate glass without a SiO layer has a surface tension of 72 mN/m, GPMVs did not spread.

Supplementary Table 2 | Surface tension (γ_s) of different substrates. Contact angles of a droplet of H_2O, I_2CH_2, or glycerin were measured (n = 3) to determine the surface tension γ_s. γ_s was calculated using the Owens Wendt Rabel Kaelble (OWRK) model. Optimum GPMV spreading was only achieved if $\gamma_s \geq 72$ mN/m, which was obtained with O_2-plasma activation for SiO_2 wafers and soda lime glass (†) or with the treatment of the SiO layer on borosilicate glass (∗) with hot water (T = 80 °C for 30 min). Errors are given as s.d.

	Surface tension (γ_s) in mN/m				
Substrate	blank	vacuum (p = 0.2 mbar)	O_2-plasma (E_{out} = 13.7 J)	SiO layer (30 nm)	activated SiO layer (30 nm)
SiO_2 wafer	64 ± 2	51 ± 1	72.0 ± 0.1†	--	--
Soda-lime glass	72.0 ± 0.3	69 ± 1	72.0 ± 0.1†	--	--
Borosilicate glass	47 ± 2	43 ± 5	72.0 ± 0.1	59 ± 6	72 ± 2∗

Another parameter that appears to be decisive is the surface roughness. Borosilicate glass has a larger surface roughness than silicon dioxide wafers, onto which GPMVs spread. On a length scale of > 250 nm, the roughness is also larger than that of soda lime glass (Supplementary Fig. 5). By evaporating 30 nm of SiO, the surface roughness is significantly reduced and does not change much even after hot water treatment, which is required to obtain the large surface tension of $\gamma_s \geq 72$ mN/m (Supplementary Fig. 5, Supplementary Table 2). Hot water treatment converts the SiO surface into a SiO_x ($1 < x < 2$) surface[4].

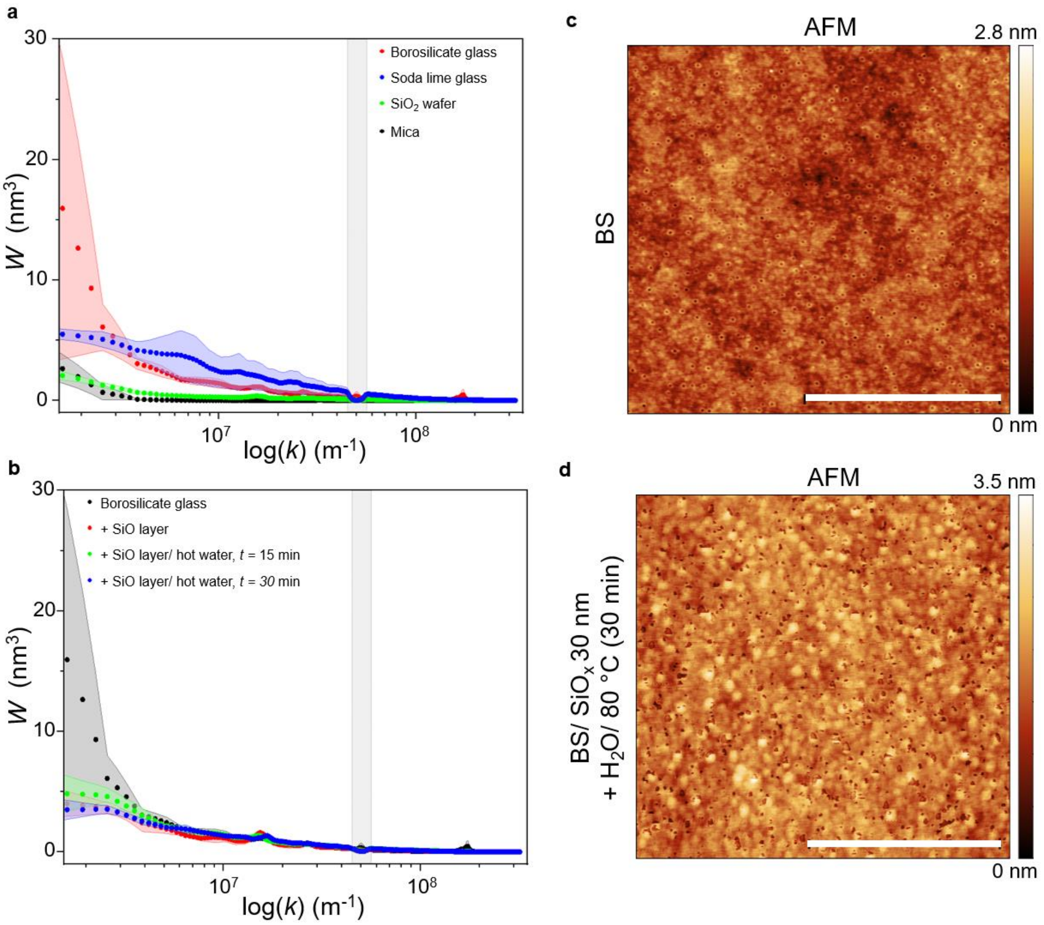

Supplementary Fig. 5 | Roughness determination of the substrates. a, Two-dimensional power spectral density functions (PSDF) for different substrates obtained from AFM images (10 × 10 µm^2; software: Gwyddion v2.56). Compared to mica and silicon wafers, borosilicate glass (BS) shows a much larger surface roughness. On a length scale of > 250 nm, the roughness is considerably larger than that of soda lime glass. **b,** After evaporation of SiO, the surface roughness is significantly reduced and does not change after hot water treatment. AFM micrographs of **c,** neat borosilicate glass, and **d,** after evaporation of a 30 nm SiO layer followed by hot water (*T* = 80 °C for 30 min). Scale bars: **c, d**: 2 µm.

Supplementary References

1 Sun, X. L. *et al.* Physical and functional interaction of Snapin with Cav1.3 calcium channel impacts channel protein trafficking in atrial myocytes. *Cell Signal* **30**, 118-129 (2017). https://doi.org:10.1016/j.cellsig.2016.11.019

2 Vivas, O., Moreno, C. M., Santana, L. F. & Hille, B. Proximal clustering between BK and $Ca_V1.3$ channels promotes functional coupling and BK channel activation at low voltage. *Elife* **6**, e28029 (2017). https://doi.org:10.7554/eLife.28029

3 Sahu, G. *et al.* Junctophilin proteins tether a Cav1-RyR2-KCa3.1 tripartite complex to regulate neuronal excitability. *Cell Rep* **28**, 2427-2442 (2019). https://doi.org:10.1016/j.celrep.2019.07.075

4 Teske, N. *et al.* Continuous pore-spanning lipid bilayers on silicon oxide-coated porous substrates. *Langmuir* **33**, 14175-14183 (2017). https://doi.org:10.1021/acs.langmuir.7b02727
